# Exploring the Relationship Between Self-Compassion and Compassion for Others: The Role of Psychological Distress and Wellbeing

**DOI:** 10.1177/10731911231203966

**Published:** 2023-10-15

**Authors:** Javier García-Campayo, Alberto Barceló-Soler, David Martínez-Rubio, Jaime Navarrete, Adrián Pérez-Aranda, Albert Feliu-Soler, Juan V. Luciano, Ruth Baer, Willem Kuyken, Jesus Montero-Marin

**Affiliations:** 1 Institute for Health Research of Aragon (IIS Aragon), Zaragoza, Spain; 2 University of Zaragoza, Spain; 3 Navarra Medical Research Institute (IdiSNA), Pamplona, Spain; 4 Universidad Europea de Valencia, Spain; 5 Teaching, Research & Innovation Unit, Parc Sanitari Sant Joan de Déu, Sant Boi de Llobregat, Spain; 6 Consortium for Biomedical Research in Epidemiology & Public Health (CIBER Epidemiology and Public Health—CIBERESP), Madrid, Spain; 7 Autonomous University of Barcelona, Spain; 8 University of Oxford, UK

**Keywords:** Sussex-Oxford Compassion Scales, Compassion Scale, Self-Compassion Scale, self-compassion, compassion for others, psychological distress, wellbeing

## Abstract

We addressed construct validity and explored the relationship between self-compassion and compassion for others using the two main current operationalizations of compassion (Neff’s and the Sussex-Oxford Compassion Scales, SOCSs). Relationships with psychological distress and wellbeing, and potential differences in the association between self-compassion and compassion for others by level of psychological distress and wellbeing were also explored. Participants (*n* = 811) completed the Spanish adaptations of the Self-Compassion Scale (SCS), the Compassion Scale (CS), the SOCSs (for the self/others), the Short Warwick–Edinburgh Mental Well-Being Scale (SWEMWBS), and the Depression Anxiety Stress Scales-21 (DASS-21). We fitted bifactor models to estimate the general factor of each construct for the different operationalizations, and calculated correlations between them. Relationships between self-compassion and compassion for others from the same operationalization were intermediate, while those between the same constructs from different operationalizations were large. Both constructs showed positive associations with wellbeing, while only self-compassion was associated with decreased psychological distress. Participants with good mental health showed higher associations between self-compassion and compassion for others than those with poorer mental health. Self-compassion and compassion for others appear to be dimensional constructs that can converge or diverge. When they converge, it is associated with better mental health.

## Introduction

Compassion is an important human capacity to respond to suffering and has been demonstrated to be associated with decreased anxious and depressive symptomatology, improved coping with pain, and positive outcomes of quality of life and wellbeing (A. López et al., 2018; Mongrain et al., 2011; Van Dam et al., 2011). Many psychological interventions either implicitly or explicitly (i.e., compassion-based psychotherapies) cultivate compassion, and changes in compassion appear to mediate changes in mental health and wellbeing (Galante et al., 2014; Navarro-Gil et al., 2020).

Compassion is central to many spiritual traditions, including Buddhism, Christianity, Hinduism, Judaism, and Islam. Although it can take different forms, the notion that compassion can be trained and cultivated, as well as the intention to transcend self-centered concerns and the invitation to respond in a friendly manner to pain and suffering, is present in all of them (Feldman & Kuyken, 2019). Compassion can be traced in our evolutionary lineage as a strong instinct that builds social groups, and allow to include those individuals who are vulnerable (Darwin, 1871). It has been described from ethology through different layers, such as the recognition of suffering, concern for others, perspective taking, and targeted helping (De Waal, 2009). Compassion and empathy are often used interchangeably, but they are distinct concepts. While empathy is the cognitive ability to understand and share the feelings of others (Davis, 1983; Decety & Jackson, 2004; Singer & Lamm, 2009), compassion involves taking action to alleviate their suffering, as it is an understanding imbued with intention (Gilbert, 2010; Goetz et al., 2010; Keltner & Kogan, 2018). Thus, while empathy can be a precursor to compassion, compassion requires the additional step of being motivated to help.

In Buddhism, compassion is one of the four qualities of mind and heart that lead to a happy and fulfilling life (Nyanaponika & Bodhi, 1999). Compassion is the wish that all beings be free from suffering and the willingness to help alleviate that suffering. Buddhism also emphasizes the importance of self-compassion, which is the practice of treating oneself with kindness, understanding, and acceptance. This value is rooted in the assumption that to have true compassion for others, one must first cultivate compassion for oneself. The practice of self-compassion involves treating oneself as one would treat a good friend, acknowledging one’s own suffering without judgment, and offering oneself kindness and support (Neff, 2003). In modern times, these concepts have been adapted into secular practices, such as mindfulness-based programs and compassion training, which have been shown to improve wellbeing, reduce stress and anxiety, and increase resilience (Hofmann et al., 2011; Neff & Germer, 2013).

Surprisingly, only recently have operational definitions of compassion been developed, and this work is still evolving. Some definitions emphasize the elements of recognition, emotional connection, and desire to alleviate suffering (Jazaieri et al., 2013); others highlight distress tolerance (Gilbert, 2009), or the acknowledgment of suffering as a universal, human experience (Feldman & Kuyken, 2011; Neff, 2003). Strauss et al. (2016) have systematically reviewed the literature and developed a comprehensive theoretical conceptualization of compassion, considering it as a cognitive, affective, and behavioral process that consists of five main elements with empirical support (Gu et al., 2017): (a) recognizing suffering; (b) understanding the universality of suffering in human experience; (c) feeling for the person suffering and emotionally connecting with their distress; (d) tolerating any uncomfortable feelings aroused in response to the suffering, so that, we remain accepting and open to the person suffering; and (e) being motivated to act to alleviate the suffering.

These five dimensions can be framed theoretically as an unfolding process that broadly speaking starts with recognition and progresses to action. They draw on contemporary commentators (Feldman & Kuyken, 2011; Gilbert, 2014) to suggest that the processes may be similar for compassion oriented toward others (i.e., compassion for others) and for compassion oriented to the self (i.e., self-compassion). However, not everyone agrees with this. For example, compassion for others has been understood as an emotion that arises when witnessing another’s suffering and that subsequently motivates a desire to help (Goetz et al., 2010), while self-compassion has also been conceptualized as a system of attitudes toward our own suffering that can present different characteristics, such as harshness or kindness, a sense of isolation or the identification of suffering as a common human experience, and a tendency to overidentify oneself with suffering or to maintain a balanced awareness (Neff, 2003). Testing these premises relies on having adequate measures of compassion, both for oneself and others.

There is a range of questionnaires designed to measure self-compassion and compassion for others, but the systematic review conducted by Strauss et al. (2016) concluded that most presented weak psychometric properties. One instrument that showed at least fair quality (i.e., 7 points of 14; Strauss et al., 2016) was the Self-Compassion Scale (SCS; Neff, 2003), a 26-item tool that is widely used and has been translated into several languages (Neff et al., 2019), including Spanish (Garcia-Campayo et al., 2014). The SCS includes six subscales (self-kindness, self-judgment, common humanity, isolation, mindfulness, and over-identification), but an overarching score of self-compassion that represents the general construct has also been proposed and evaluated using bifactor exploratory structural equation modeling (ESEM) (Neff et al., 2019). The same authors have developed the Compassion Scale (CS; Pommier et al., 2020), a 16-item instrument to assess compassion for others based on Neff’s theoretical conceptualization of self-compassion (Neff, 2003). The CS operationalizes compassion as experiencing kindness, a sense of common humanity, mindfulness, and lessened indifference toward the suffering of others. This four-dimensional operationalization of compassion for others that integrates all the negative items into only one factor has originally presented sound psychometric properties using bifactor ESEM, but it has not been widely used to date, and there is not a validated Spanish version of this scale.

In response to the need for theoretically comprehensive and psychometrically robust operational measures of both self-compassion and compassion for others, Gu et al. (2020) developed the Sussex-Oxford Compassion Scales (SOCSs) based on the multidimensional conceptualization proposed by Strauss et al. (2016). These scales assess compassion for others (SOCS-O), and compassion for the self (SOCS-S), both along the five dimensions of compassion mentioned above. It has been suggested that although a total score can be derived from both the SOCS-O and SOCS-S using a five-factor hierarchical model that has shown adequate fit in samples of health care workers and university students from the United Kingdom, as these scales were originally designed to be multidimensional, it would be important to examine its potential one-dimensionality in greater detail, and how their dimensions collectively relate to outcomes (Gu et al., 2020). In addition, some psychometric properties, for example, test–retest reliability, remain unexplored. SOCS-O scores were higher in females, in line with A. López et al. (2018), and in people with previous meditation experience; the latter finding was expected because compassion is a core element of many contemplative programs (Jahoda et al., 2017). Correlations between the SOCS-O and the SOCS-S were significant and moderate, but this finding needs to be further tested and cross-validated in other populations (Gu et al., 2020).

Some studies have reported small but significant positive correlations between compassion for the self and compassion for others (Neff & Pommier, 2013; Pommier et al., 2020). It has also been found that activating support-giving schemas increased self-compassion (Breines & Chen, 2013), and that similar brain regions were activated both when expressing self-reassurance and compassion for others (Longe et al., 2010). However, some studies have also reported no significant associations between self-compassion and compassion for others. Leary et al. (2007) found that compassion for others was not significantly different among individuals who presented low vs. high self-compassion. Specifically, they saw in undergraduate students that self-compassion predicted positive affect when participants watched a videotape of their own performance, but not when they watched others’ tapes, suggesting that self-compassion might be distinct from more general feelings of compassion for others. A. López et al. (2018) found no significant relationships between self-compassion and compassion for others in a community sample of adults, and reported that sociodemographic factors, such as gender and education level, were differently associated with each type of compassion: compassion for others was higher in women and in people with lower levels of education, while self-compassion was lower in people with lower levels of education. Durkin et al. (2016) observed no significant relationships between compassion for others and self-compassion in community nurses. Therefore, the association between self-compassion and compassion for others, remains unclear.

There is a natural motivation to seek care and connect with others that is thought to be an evolved trait that has helped build social groups and promote survival in our evolutionary history. However, when we are struggling with mental health issues, this natural motivation to seek care and connect with others may become distorted or inhibited (Gilbert et al., 2017). In general, it has been observed that individuals tend to display more compassion to others than to themselves (Neff, 2003). However, in populations with high levels of mental distress, there may be a wide variation in the ways self-compassion and compassion for others are manifested (Gilbert et al., 2017). Poor mental health may be related to distortions of our natural inclination to connect with care-seeking experiences from ourselves and others. For example, an individual suffering from trauma might display compassion for others but may be filled with self-criticism and unable to receive compassion from others (Montero-Marín et al., 2016; Van der Kolk, 2014). It has been proposed that this distortion of our innate motivational system may be a contributing factor to poor mental health outcomes. Therefore, by recognizing and addressing this distortion, we may be better able to promote mental wellbeing and improve our ability to connect with others in a healthy and supportive way (Gilbert, 2022).

In this context, we first adapted to the Spanish population and tested the construct validity of the two main current operationalizations of compassion—Neff’s model and the SOCSs model (Gu et al., 2020; Neff, 2003; Pommier et al., 2020)—to see whether they are adequate measures of compassion. We expected the new Spanish operationalizations to have a similar structure to those observed in their original English validations (Gu et al., 2020; Neff, 2003; Pommier et al., 2020).

Once we established the measurement models for the different constructs and operationalizations of compassion, we attempted to determine the extent to which self-compassion and compassion for others converge. For that, we calculated the associations between the CSs in the same operationalization (SOCS-O/SOCS-S; CS/SCS) and between those compassion scales that measure the same construct in the different operationalizations (SOCS-O/CS; SOCS-S/SCS). Our exploratory hypothesis is that relationships between scales that measure the same compassion construct using different operationalizations will be stronger than those that measure different compassion constructs through the same operationalization, and therefore reflecting potential different modes (i.e., differences in terms of a higher or lower aggregation) of the motivational system functioning (Gilbert et al., 2017).

We additionally assessed the explanatory power of both self-compassion and compassion for others in accounting for variance in psychological distress and mental wellbeing, and examined whether the relationship between self-compassion and compassion for others is modified by levels of psychological distress and mental wellbeing. Previous research (Neff & Germer, 2013) has shown that self-compassion (in short, as an attempt to soothe distress in oneself) is a stronger predictor of mental health than compassion for others. Thus, we expected self-compassion to be more correlated than compassion for others with both psychological distress (in a negative way) and wellbeing (in a positive way). Finally, we hypothesized that the relationship between self-compassion and compassion for others will be stronger in individuals with lower psychological distress and higher mental wellbeing, indicating a more aggregated self-compassion–compassion for others motivational structure (Gilbert, 2022).

We carried out all of these explorations using two different and concurrent operationalizations of compassion, allowing us to determine whether the lack of consistency in previous studies (Gu et al., 2020; Leary et al., 2007; A. López et al., 2018; Pommier et al., 2020) could be due to the use of different operational definitions.

## Methods

A cross-sectional internet survey was disseminated through several websites from the authors’ scientific research webpage. It was carried out on a commercial system (https://es.surveymonkey.com) using a series of questionnaires that were presented in a random order every time they were to be completed to control for carry-over effects (e.g., missing data, less accuracy due to fatigue, etc.).

### Data Collection, Participants, and Ethics

**Figure 1.**
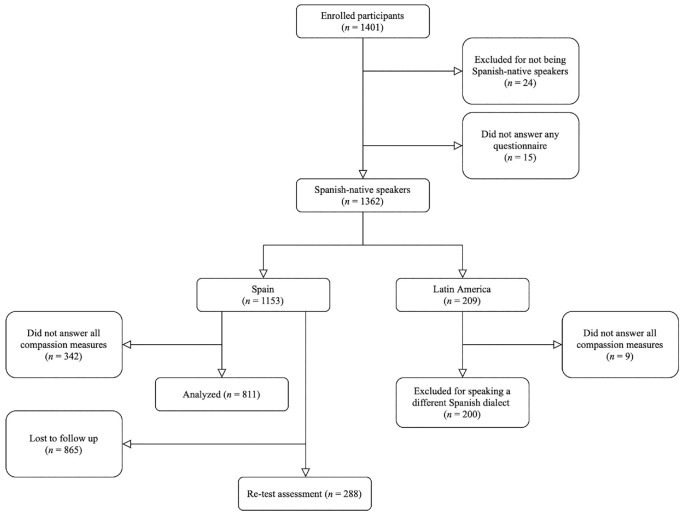
Study Flowchart.

The recruitment of participants was carried out through the social networks of the research group. Individuals were invited to participate in a study on “general aspects of compassion.” The information that appeared in the study announcement was the main objective, the link to the online questionnaire, and an email address where participants could contact in case of any doubt about the study. To control for the risk of responses generated by bots, and so on, the IP address and completion time of each participant were recorded by the online platform and reviewed by a research assistant. The link to the survey was accessible from June 2021 to December 2021. A total of 1,401 individuals accessed the link and voluntarily agreed to participate in the online survey (Figure 1). Among them, 1,362 were Spanish native speakers, with 1,153 having Spanish nationality and 209 being from Latin America. Finally, 811 Spanish native speakers with Spanish nationality (Table 1) completed all SOCS-O, SOCS-S, CS, and SCS items and thus configured the analytic complete case sample (pairwise deletion was the technique used to handle missing data). Most participants were female (80.0%), with a mean age of 43.49 (*SD* = 12.8; range = 18–85), and mainly married (53.0%), with no children (48.3%), a university education (82.3%), and in employment (66.6%). A subsample of 288 (35.5%) individuals completed the SOCS-O and SOCS-S 1 week later to evaluate the stability over time (Supplemental Table S1). The characteristics of Spanish native speakers who accessed but did not complete the survey can be seen in Supplemental Table S2.

**Table 1.** Sociodemographic Data of the Study Participants (n = 811).

Gender (women): *n* (%)	649 (80.0)
Age (in years): *M* (*SD*)	43.49 (12.8)
Marital status: *n* (%)	
Single	282 (34.8)
Married/civil partner	430 (53.0)
Separated/divorced	81 (10.0)
Widowed	18 (2.2)
Children: *n* (%)	
0	392 (48.3)
1	139 (17.2)
2	230 (28.4)
3	40 (4.9)
4	10 (1.2)
Level of education: *n* (%)	
No schooling	2 (0.2)
Primary school	46 (5.7)
Secondary school	96 (11.8)
University	667 (82.3)
Work status: *n* (%)	
Student	108 (13.3)
Unemployed	44 (5.4)
Employed	540 (66.6)
Self-employed	32 (4.0)
Homemaker	9 (1.1)
On a sick leave	23 (2.8)
Retired/pensioner	43 (5.3)
Unable to work	12 (1.5)

*Note. n* = frequencies. % = percentages.

We carried out post hoc power analyses to evaluate the viability of a bifactor exploratory structure (Morin et al., 2016) as a measurement model for the SOCS-O, SOCS-S, CS, and SCS (Supplemental Figure S1). This measurement model permits us to represent the general constructs mentioned underlying the corresponding scales, allowing the coexistence of the general factors and their subdomains, and including the possibility that items may present cross-loadings on the nontarget subdomains, thus estimating the true variance of the general dimension (Morin et al., 2016). This approach represents the most comprehensive measurement model that can accurately describe complex psychological characteristics, such as those under study, facilitating their comparability. Details on the statistical procedures that were used to evaluate the viability of this measurement model’s application to the CSs are in Supplemental Table S3. To test this, a sample of *n* = 811 subjects, with a null hypothesis that root mean square error of approximation (Root Mean Squared Error of Approximation [RMSEA]) would be ≤ .05 if the true value was .08 (close fit) and an alpha equal to .05, produces a power coefficient (1-beta) in all the models of .99 (MacCallum et al., 1996).

The study protocol was approved by the Ethical Committee of the regional health authority of Aragon (CEICA, PI21/197; 05-May-2021). All participants submitted a written informed consent form online attesting to their willingness to participate.

### Measures

#### Sociodemographic Characteristics

We collected information about self-identified gender (male and female), age, marital status (single, married/civil partner, separated/divorced, and widowed), number of children, education level (no schooling, primary school, secondary school, and university), and work status (student, unemployed, employed, self-employed, homemaker, and retired/pensioner).

#### Compassion for the Self/Compassion for Others

##### Sussex-Oxford Compassion Scales

Permission from the original authors was obtained for translating/validating the Spanish version of the SOCSs (Gu et al., 2020). A description of the adaptation process and the SOCSs Spanish versions are shown in Supplemental Tables S4–S6. The SOCS-S and SOCS-O are two 20-item self-report measures that contain five subscales (four items for each): recognizing suffering, understanding the universality of suffering, feeling for the person suffering, tolerating uncomfortable feelings, and acting or being motivated to act to alleviate suffering. Items are scored on a 5-point Likert-type scale, ranging from 1 (“not at all true”) to 5 (“always true”). The higher the score is, the greater the degree of self-compassion/compassion for others. The bifactor exploratory measurement model presented adequate fit indices for both the SOCS-S (comparative fit index [CFI] = .995, Tucker–Lewis index [TLI] = .988, RMSEA = .054; 90% confidence interval [CI] = .043–.065, weighted root mean square residual [WRMR] = .418) and SOCS-O (Comparative fit index [CFI] = .995, Tucker-Lewis index [TLI] = .988, RMSEA = .054; 90% Confidence Interval [CI] = .043–065, Weighted Root Mean square Residual [WRMR] = .418). The internal consistency of the general factor (omega hierarchical, ω_h_) was good (SOCS-O: ω_h_ = .86, SOCS-S: ω_h_ = .87). The descriptive statistics of the SOCSs, factor loadings, and other reliability indices can be found in Supplemental Tables S7–S9. An exploration of the fit of the other potential measurement models can be seen in Supplemental Table S10.

##### Self-Compassion Scale

The SCS (Neff, 2003) is a 26-item self-report measure of the following six constituent elements of self-compassion (Neff, 2016): self-kindness (five items), common humanity (four items), mindfulness (four items), self-judgment (five reversed scored items), isolation (four reversed scored items), and over-identification (four reverse scored items). The scale has a 5-point Likert-type response format ranging from 1 (“almost never”) to 5 (“almost always”), with higher scores indicating higher self-compassion levels. The validated Spanish version of the SCS was used (Garcia-Campayo et al., 2014). The Spanish SCS version is available in Supplemental Table S11. A previous study proposed the bifactor exploratory measurement model as the best representation structure for the SCS (Neff et al., 2019). The descriptive statistics of the SCS and factor loadings using the bifactor exploratory measurement model can be found in Supplemental Table S12. This model presented good fit indices for the SCS (CFI = .989, TLI = .979, RMSEA = .051; 90% CI = .044–.059, WRMR = .446). The internal consistency of the general factor was good (ω_h_ = .92). Other reliability indices can be found in Supplemental Table S13. An exploration of the fit of other potential measurement models can be seen in Supplemental Table S14.

##### Compassion Scale

Permission from the original authors was obtained for translating/validating the Spanish version of the CS (Pommier et al., 2020). A description of the adaptation process and the CS Spanish version are available in Supplemental Tables S4 and S15. The CS is a 16-item Likert-type scale with a 5-point response format ranging from 1 (“almost never”) to 5 (“almost always”) that assesses compassion for others. It is based on the SCS model (Neff, 2003) and considers four constituent elements: kindness (four items), common humanity (four items), mindfulness (four items), and indifference (four reverse scored items). Higher scores indicate higher compassion for others. The bifactor exploratory measurement model has been proposed as the best representation structure of the CS (Pommier et al., 2020). It presented adequate fit indices in this study (CFI = .99, TLI = .98, RMSEA = .04; 90% CI = .03–.05, WRMR = .52), and the internal consistency of the general factor was good (ω_h_ = .78). The descriptive statistics, factor loadings, and other reliability indices of the CS can be found in Supplemental Tables S16 and S17. An exploration of the fit of other potential measurement models can be seen in Supplemental Table S14.

#### Psychological Distress and Mental Wellbeing

##### Depression Anxiety Stress Scale-21

The Depression Anxiety Stress Scale-21 (DASS-21; Lovibond & Lovibond, 1995) is a 21-item self-report measure composed of three seven-item subscales that assess depression (e.g., “I felt that I had nothing to look forward to”), anxiety (e.g., “I felt I was close to panic”), and stress (e.g., “I tended to overreact to situations”). It has a 4-point Likert-type response format from 0 (“did not apply to me at all”) to 3 (“applied to me very much or most of the time”). A total score is computed by summing all the items, with a higher score indicating higher psychological distress (range: 0–63). The validated Spanish version of the DASS-21 was used (Bados et al., 2005), which showed good internal consistency in the present study (composite reliability, ω = .94).

##### Short Warwick–Edinburgh Mental Well-Being Scale

The Short Warwick–Edinburgh Mental Well-Being Scale (SWEMWBS; Stewart-Brown et al., 2009) is a seven-item measure of mental wellbeing (e.g., “I’ve been dealing with problems well”), with a 5-point Likert-type scale from 1 (“none of the time”) to 5 (“all of the time”). The time frame of the instrument was the last 2 weeks. An overall score for the SWEMWBS can be calculated by summing the scores of each item (ranging from 7 to 35). The items are worded positively, and thus a higher score indicates higher mental wellbeing levels. Items from the Spanish version of the SWEMWBS (M. A. López et al., 2013) were used, which showed adequate internal consistency values in the present study (ω = .84).

### Data Analysis

Factor structure and reliability of the Spanish versions of the SOCSs, SCS, and CS were assessed first (Supplemental Figure S1 and Table S3). Then, we computed the correlation analyses to explore relationships between the general factors that measure the different constructs (i.e., self-compassion vs. compassion for others) from the same operationalization, and the relationships between those general factors that measure the same construct from the different operationalizations (Neff’s model vs. SOCS’s model). For that, we used total factor scores that were calculated by means of bifactor exploratory measurement models (Supplemental Figure S1) and Pearson’s correlation coefficients, as well as partial correlations after controlling for the general sociodemographic characteristics (e.g., gender, age, marital status, number of children, education level, and work status). Total factor scores were used because they are better proxies of complex latent variables than raw sum scores (T. D. Brown, 2015). The strengths of the associations were interpreted as follows (Cohen, 1988): *r* = .10 to .29 (small); *r* = .30 to .49 (medium); *r* = .50 to 1 (large).

To assess the explanatory power of both self-compassion and compassion for others in accounting for variance in psychological distress and mental wellbeing, we first calculated Pearson’s correlation coefficients between total factor scores for self-compassion and compassion for others, using the different operationalizations, on one hand, and for psychological distress and wellbeing, on the other hand. Second, we calculated partial correlations and multivariable linear regressions predicting psychological distress and mental wellbeing using both constructs and Neff’s and SOCS operationalizations separately controlling for the sociodemographic variables.

We explored potential differences in the association between self-compassion and compassion for others by level of psychological distress and wellbeing. For that, the sample was split according to a 16 total score in the DASS-21, that is, the cut-off score for screening anxiety or major depression (Chin et al., 2019), and a 28 total score in the SWEMWBS, which has been proposed to identify participants with high levels of wellbeing (Ng et al., 2017). We described the distribution of self-compassion and compassion for others by subgroup using means (*SD*s), and calculated effect sizes using Cohen’s *d* and 95% confidence intervals. Pearson’s correlations between self-compassion and compassion for others were calculated for each subgroup independently (partial correlations adjusting for the sociodemographic features of participants also were estimated). Then, a *z*-value was calculated using the Fisher *r*-to-*z* transformation (Fisher, 1925), and the significance of the difference between correlation coefficients was computed (Steiger, 1980).

All the tests were two-sided and were performed with a significance level of α < .05. Nevertheless, given the exploratory nature of this study, we did not correct for multiple testing but balanced statistical significance considering the amplitude of the findings (i.e., effect size), and regarded them as tentative and hypothesis generating (Feise, 2002). Data analysis was conducted using SPSS v26 and Mplus v8.4.

## Results

The raw correlations between the different constructs (i.e., self-compassion and compassion for others) from the same operationalization (i.e., Neff’s and SOCSs) were small (range: *r* = .14–.29), while the raw correlations between the same constructs from the different operationalizations were large (range: *r* = .65–.79), suggesting more convergence in terms of operationalizations than regarding constructs (Supplemental Figure S2). The same pattern of relationships was observed after controlling for the sociodemographic characteristics of the participants (Table 2).

**Table 2.** Partial Correlations Between the Two Compassion Operationalizations Controlling for the Sociodemographic Characteristics of Participants (n = 811).

Measure	SOCS-O		CS		SOCS-S	
	*R*	*p*	*r*	*p*	*r*	*p*
CS	.64	< .001				
SOCS-S	.29	< .001	.32	< .001		
SCS	.19	< .001	.27	< .001	.78	< .001

*Note.* Partial correlation coefficients were calculated by controlling for the following sociodemographic characteristics: sex, age, marital status, number of children, education level, and work status. SOCS-O = Sussex-Oxford Compassion Scale-Others; CS = Compassion Scale (others); SOCS-S = Sussex-Oxford Compassion Scale-Self; SCS = Self-Compassion Scale; *r* = partial correlation coefficient; *p* = *p* value.

The raw relationships between compassion for others (using both Neff’s and SOCS operationalizations), and psychological distress and wellbeing were small although significant and in the expected direction (range *r* = .09–.26 in absolute value). The raw relationships between self-compassion (using both Neff’s and SOCS operationalizations), and psychological distress and wellbeing were medium to large, and they also were positive and significant (range *r* = .48 and .59 in absolute value) (Supplemental Figure S3). However, after controlling for the sociodemographic characteristics of participants, the relationships between compassion for others, using both Neff’s and SOCS operationalizations, and psychological distress were very small, and they did not reach statistical significance (Table 3). Details of a hierarchical multivariable linear regression analysis predicting psychological distress and wellbeing using both constructs and Neff’s and SOCS operationalizations separately after controlling for the sociodemographic variables can be found in Tables S18–S21.

**Table 3.** Partial Correlations of Self-Compassion/Compassion for Others and Psychological Distress/Mental Wellbeing Controlling for the Sociodemographic Characteristics of Participants (n = 811).

	DASS-21		SWEMWBS	
	*r*	*p*	*R*	*p*
Compassion for others				
SOCS-O	–.01	.909	.10	.005
CS	–.07	.057	.17	< .001
Compassion for the self				
SOCS-S	–.43	< .001	.54	< .001
SCS	–.52	< .001	.54	< .001

*Note.* Partial correlation coefficients were calculated by controlling for the following sociodemographic characteristics: sex, age, marital status, number of children, education level, and work status. DASS-21 = Depression Anxiety Stress Scale-21; SWEMWBS = Short Warwick–Edinburgh Mental Well-Being Scale; SOCS-O = Sussex-Oxford Compassion Scale-Others; CS = Compassion Scale (others); SOCS-S = Sussex-Oxford Compassion Scale-Self; SCS = Self-Compassion Scale; *r* = Partial correlation coefficient; *p* = *p* value.

The distribution of the compassion measures by subgroup can be seen in Supplemental Table S22. The high wellbeing subgroup (*n* = 354), compared with the low wellbeing subgroup (*n* = 457), showed higher scores in all the compassion measures, with small-to-medium effects for the SOCS-O (*d* = 0.37), medium effects for the CS (*d* = 0.53), and large effects for the SOCS-S and SCS (*d* = 1.13 and *d* = 1.15, respectively). The low psychological distress subgroup (*n* = 538), compared with the high psychological distress subgroup (*n* = 273), showed higher scores in all the compassion measures but SOCS-O, with very small effects for the SOCS-O (*d* = 0.08), small effects for the CS (*d* = 0.28), and large effects for the SOCS-S and SCS (*d* = 0.87, and *d* = 1.03, respectively). Differences between subgroups were significantly higher in self-compassion that in compassion for others, using both Neff’s and SOCS operationalizations. The raw correlations between self-compassion and compassion for others were small to intermediate in the subgroup of low psychological distress (range *r* = .20–.33), and they were significantly larger than in the subgroup of high psychological distress, in which they were null to small (range *r* = .02–.14) (Supplemental Table S22). The same pattern of relationships was found after controlling for the sociodemographic characteristics (Table 4). The raw correlations between self-compassion and compassion for others were small to intermediate in the subgroup of high wellbeing (range *r* = .23–.33), and they were significantly larger than in the subgroup of low wellbeing, in which they were null (range *r* = –.08 to .09) (Supplemental Table S22). An almost identical pattern was found after controlling for the sociodemographic features (Table 4).

**Table 4.** Partial Correlations (Controlling for the Sociodemographic Characteristics of Participants) Between Self-Compassion and Compassion for Others Per Psychological Distress and Wellbeing Subgroup Status, and Differences Between Correlations.

	CS		SOCS-S		SCS	
	*r*	*p*	*r*	*p*	*r*	*p*
DASS-21 ≤ 16 (*n* = 538)						
SOCS-O	.66	.000	.35	.000	.25	.000
CS			.38	.000	.30	.000
SOCS-S					.76	.000
DASS-21 > 16 (*n* = 273)						
SOCS-O	.60	.000	.16	.009	.04	.490
CS			.12	.051	.09	.176
SOCS-S					.70	.000
	*z*	*p*	*z*	*p*	*z*	*p*
DASS-21 ≤ 16 vs. DASS-21 > 16						
SOCS-Others	1.33	.182	2.73	.006	2.88	.004
CS			3.74	.000	2.93	.003
SOCS-Self					1.72	.085
	CS		SOCS-S		SCS	
	*r*	*p*	*r*	*p*	*r*	*p*
SWEMWBS ≥ 28 (*n* = 354)						
SOCS-O	.66	.000	.34	.000	.25	.000
CS			.34	.000	.30	.000
SOCS-S					.76	.000
SWEMWBS < 28 (*n* = 457)						
SOCS-O	.58	.000	.11	.018	-.04	.438
CS			.11	.016	.04	.469
SOCS-S					.69	.000
	*z*	*p*	*z*	*p*	*z*	*p*
SWEMWBS ≥ 28 vs. SWEMWBS < 28						
SOCS-Others	1.83	.067	3.43	.000	3.03	.002
CS			3.43	.000	3.79	.000
SOCS-Self					2.08	.037

*Note.* Partial correlation coefficients were calculated by controlling for the following sociodemographic characteristics: sex, age, marital status, number of children, education level, and work status. DASS-21 = Depression Anxiety Stress Scale-21; SWEMWBS = Short Warwick–Edinburgh Mental Well-Being Scale; SOCS-O = Sussex-Oxford Compassion Scale-Others; CS = Compassion Scale (others); SOCS-S = Sussex-Oxford Compassion Scale-Self; SCS = Self-Compassion Scale; *r* = partial correlation coefficient; *z* = Steiger’s z for significance of the difference between correlations.

## Discussion

This research is innovative in several ways. First, it addresses the construct validity and reliability of the two main current operationalizations of compassion for the Spanish population, which has not been done before for some of them (e.g., SOCS-S, SOCS-O, CS). Second, the study examines the extent to which self-compassion and compassion for others converge, and this had not been explored in previous research using two different operationalizations concurrently. Third, the research also explores the associations between self-compassion and compassion for others in relation to psychological distress and mental wellbeing, and specifically investigates whether the relationship between self-compassion and compassion for others is influenced by levels of psychological distress and mental wellbeing. This aspect has not been adequately studied in previous research. The use of two different operationalizations of compassion concurrently allows for a comparison of results and an evaluation of the consistency of previous studies. Overall, this research provides new insights into the construct of compassion, its measurement, and its relationship with mental health outcomes.

We explored the relationship between self-compassion and compassion for others using two different operationalizations of compassion: Neff’s and the SOCSs models (Gu et al., 2020; Neff, 2003; Pommier et al., 2020). But first, we reproduced all the factorial structures that were originally proposed (i.e., one-factor model, correlated factors model, hierarchical model), and also evaluated the bifactor model following a recent proposal for the Neff’s operationalization (Neff et al., 2019), and extended it to the SOCSs to evaluate its potential unidimensionality in more detail. Strong general factors of self-compassion and compassion for others in both operationalizations were observed, suggesting a possible unidimensional (although multifaceted) nature of these constructs, allowing the use and comparison of overall scores under similar metric conditions. Nevertheless, it has been proposed that the extent to which the positive and negative items of the SCS contribute to explain self-compassion, and the possibility of different self-compassion facets being formed by positive and negative items as reflecting a broader construct, might differ across cultural backgrounds (Montero-Marin et al., 2018). It has been also referred that the use of negative items (as it occurs in the SCS, and CS) is not in agreement with the protective nature of the compassion construct, and that a clear understanding of compassion would need to separate the influence of negative components to avoid its potential tautological influence (Muris et al., 2016). This was one of the premises of the SOCSs operationalizations, which only use positive items.

Previous studies have reported significant associations between self-compassion and compassion for others, although modest in most cases (Ari et al., 2022; Gu et al., 2020; Neff & Pommier, 2013; Pommier et al., 2020; Tendhar et al., 2022), but some studies have found small and no significant relationships (Durkin et al., 2016; Leary et al., 2007; A. López et al., 2018). These inconsistent findings may be at least in part due to issues with the previous measures of compassion used. Our findings considering the general factors of self-compassion and compassion for others derived from both Neff’s and SOCSs’ operationalizations support the hypothesis that self-compassion and compassion for others are different yet related constructs, since the relationships between the scales measuring the same construct (i.e., SOCS-O/CS; SOCS-S/SCS, with large effects) were larger than the associations found between the scales of each operationalization (i.e., SOCS-O/SOCS-S; CS/SCS, with small effects), even after controlling for general sociodemographic characteristics.

Self-compassion was strongly correlated with both psychological distress and wellbeing, and presented high explanatory power on these variables. This finding is in accordance with previous evidence, since self-compassion has been consistently linked to less psychological distress and higher wellbeing (Marsh et al., 2018). The meta-analysis conducted by Zessin et al. (2015) observed a strong association between them, particularly in the cases of cognitive and psychological wellbeing. On the other hand, in our study, compassion for others was not associated with psychological distress after controlling for the sociodemographic characteristics of the sample, and although significant correlations with wellbeing were observed, these associations were to a much lesser extent compared with self-compassion. Compassion for others has not been as widely studied as self-compassion, and to our knowledge, no meta-analyses have been conducted on its relationships with mental health-related outcomes, such as psychological distress or wellbeing. However, most studies seem to point out that compassion for others is not significantly correlated with these variables (Beaumont et al., 2016; A. López et al., 2018), or not as much as self-compassion (Tendhar et al., 2022). A cross-cultural study comparing Japanese and U.S. samples found that self-compassion was related to positive and negative affect, social anxiety and wellbeing in both countries, while compassion for others was associated with only positive affect and social anxiety symptoms (Arimitsu et al., 2019). A recent study aiming to reduce stress and promote wellbeing in health care workers using a mindfulness-based program observed a potential mechanistic role of self-compassion in both outcomes, but this was not observed in the case of compassion for others (Strauss et al., 2021). All these results indicate that, as our results suggest and as hypothesized, self-compassion is more strongly related to negative and also positive indicators of mental health than compassion for others.

We examined whether the relationship between self-compassion and compassion for others can differ among different levels of psychological distress and wellbeing. As expected, the correlations between compassion for the self and for others were significant and positive, with intermediate effects in the subgroups of participants with low psychological distress and/or high wellbeing, but they were significantly lower, with small or even absent effects, in the participants with high psychological distress and/or low wellbeing. This result indicates that mentally healthy individuals tend to present similar tendencies to apply compassionate attitudes toward themselves and others, indicating a more aggregated motivational structure (Gilbert, 2022). According to Gilbert’s (1989, 2009, 2022; Gilbert et al., 2017) social mentality theory, self-compassionate attitudes are enabled by having received compassion from others through secure attachment relationships, which is in turn associated with reduced stress and higher positive affect, including positive psychological wellbeing (Di Bello et al., 2020; Stellar et al., 2015), as reported in the present study. In general, individuals tend to display less self-compassion than compassion for others (Neff, 2003), and we observed this was specially so for those with worse mental health. It has been observed that psychopathology is usually accompanied by low levels of self-compassion (Athanasakou et al., 2020), while self-compassion might have a central role in recovery (Waite et al., 2015). The observed lack of associations between self-compassion and compassion for others in individuals with poor mental health may have psychological and physiological underpinnings (Hermanto & Zuroff, 2016), suggesting there are probably other relevant variables involved in this relationship, such as the potential existence of a psychiatric disorder, and its nature. The ability to experience empathy and the tendency to trust or distrust others are key aspects of some disorders (e.g., psychosis, autism, antisocial personality disorder), while these are not necessarily present in other psychopathologies, or in people with psychological distress but no psychopathology. Such tendency to experience empathy and trust/distrust others have been related to the ability to experience compassion for others (P. Brown et al., 2020; Singer & Klimecki, 2014). Thus, it could be hypothesized that while self-compassion would generally be low in most people with poor mental health (irrespective of the presence of a disorder, and its nature), their clinical profile could play a role in the levels of compassion for others, but this hypothesis should be tested in further studies.

The present work presents three notable strengths: it was conducted on a large general sample of Spanish participants; it provides a validated version of the SOCSs and CS to be used in Spanish-speaking clinical/research settings; and it evaluated the potential unidimensionality of the constructs under study that came from the different operationalizations of compassion (i.e., Neff’s and the SOCSs models) in great detail. Nevertheless, some limitations need to be acknowledged. We did not administer the translated version of our questionnaire to a bilingual sample, alongside the English version. Doing so would have enhanced confidence that the two measures function similarly. The online assessment that was conducted for this study could have implied a self-selection bias (i.e., people without digital literacy, or no access to the internet could have been excluded) (Wright, 2005). As the use of Virtual Private Networks (VPNs) is becoming relatively common, IP addresses may not accurately reflect a participant’s legitimacy to participate or location, and multiple individuals from the same household (who might be using the same VPN) could appear to have the same IP address, leading to inaccuracies in data collection. The cross-sectional nature of our study hinders the extraction of conclusions based on causality; although the impact of self-compassion on wellbeing and psychological distress can be assumed based on previous randomized controlled trials that have proven the effects of compassion-based interventions on improving these outcomes in different samples (Kirby et al., 2017), the potential causal connection between presenting low levels of self-compassion and experiencing mental disorders is still to be determined. Finally, participants were not evaluated using a clinical interview, systematic observation, or experimental tasks, and our results are solely based on self-reported measures. Future studies should include other type of assessments to both contrast the results of our study (i.e., absence of associations between self-compassion and compassion for others in people with poor mental health) and to explore the hypothesis that has been raised (i.e., such a relationship being larger in some types of disorders than in others).

New research confirming the intermediate association between self-compassion and compassion for others in the healthy population and the absence of relationships in specific subgroups suffering from psychopathology seems warranted. Future research should investigate whether the potential decoupling process between self-compassion and compassion for others in psychiatric patients can be reverted, if this is possible using different types of interventions (e.g., psychotherapies, and drugs), and the extent to which this could be a potential cause or an epiphenomenon of clinical improvements.

## Supplemental Material

sj-docx-1-asm-10.1177_10731911231203966 – Supplemental material for Exploring the Relationship Between Self-Compassion and Compassion for Others: The Role of Psychological Distress and Wellbeing Supplemental material, sj-docx-1-asm-10.1177_10731911231203966 for Exploring the Relationship Between Self-Compassion and Compassion for Others: The Role of Psychological Distress and Wellbeing by Javier García-Campayo, Alberto Barceló-Soler, David Martínez-Rubio, Jaime Navarrete, Adrián Pérez-Aranda, Albert Feliu-Soler, Juan V. Luciano, Ruth Baer, Willem Kuyken and Jesus Montero-Marin in Assessment
