# Supplementary material for: Exploring the Relationship Between Self-Compassion and Compassion for Others: The Role of Psychological Distress and Wellbeing
Source: Assessment. 2023 Oct 15;31(5):1038–51. doi: 10.1177/10731911231203966 (PMC11134997; doi:10.1177/10731911231203966)
Supplement: sj-docx-1-asm-10.1177_10731911231203966 – Supplemental material for Exploring the Relationship Between Self-Compassion and Compassion for Others: The Role of Psychological Distress and Wellbeing [file sj-docx-1-asm-10.1177_10731911231203966.docx]

**Exploring the relationship between self-compassion and compassion for others: the role of psychological distress and wellbeing**

**Supplementary Materials**

**Table S1**: Sociodemographic characteristics of the retest sample (n = 288)…………………………3

**Table S2**: Sociodemographic data of the non-completers Spanish native speakers (n = 351)……….4

**Figure S1**: Bifactor exploratory measurement models……………………………………………….5

**Table S3**: Statistical procedures used to address the construct validity, internal consistency, and test-retest validity of the measurement models for the compassion scales……………………………….6

**Table S4**: Spanish adaptation process of the SOCS-O, SOCS-S, and CS……………………………8

**Table S5**: Sussex-Oxford Compassion for Others Scale (SOCS-O) Spanish version………………..9

**Table S6**: Sussex-Oxford Compassion for the Self Scale (SOCS-S) Spanish version……………10

**Table S7**: Descriptive statistics of the SOCS-O and standardised factor loadings for the bifactor exploratory measurement model (n = 811)…………………………………………………………..11

**Table S8**: Descriptive statistics of the SOCS-S and standardised factor loadings for the bifactor exploratory measurement model (n = 811)…………………………………………………………..12

**Table S9**: Reliability indices of the bifactor exploratory measurement model for the Sussex-Oxford Compassion Scales (SOCS-S and SOCS-O)………………………………………………………...13

**Table S10**: Goodness-of-fit indices of factor models for the Sussex-Oxford Compassion Scales (SOCS-S and SOCS-O)……………………………………………………………………………...14

**Table S11**: Self-Compassion Scale (SCS) Spanish version (García-Campayo et al., 2014)……...15

**Table S12**: Descriptive statistics of the SCS and standardised factor loadings for the bifactor exploratory measurement model (n = 811)…………………………………………………………..16

**Table S13**: Reliability indices of the bifactor exploratory measurement model for the Self-Compassion Scale (SCS)…………………………………………………………………………….17

**Table S14**: Goodness-of-fit indices of the factor models for the Self-Compassion Scale (SCS) and Compassion Scale (CS)……………………………………………………………………………...18

**Table S15**: Compassion Scale (CS) Spanish version……………………………………………...19

**Table S16**: Descriptive statistics of the CS and standardised factor loadings for the bifactor exploratory measurement model (n = 811)…………………………………………………………..20

**Table S17**: Reliability indices of the bifactor exploratory measurement model for the Compassion Scale (CS)……………………………………………………………………………………………21

**Figure S2**: Pearson’s correlations between self-compassion and compassion for others using the different operationalisations (n = 811)…………………………………………….…………………22

**Figure S3**: Pearson’s raw correlations of study variables (n = 811)………………………………23

**Table S18**: Hierarchical multiple regression analysis predicting DASS-21 with ‘Sociodemographic + SOCSs’ (Step 2)………………………………………… ………………...24

**Table S19**: Hierarchical multiple regression analysis predicting SWEMWBS with ‘Sociodemographic + SOCSs’ (Step 2)……………………………………………………………25

**Table S20**: Hierarchical multiple regression analysis predicting DASS-21 with ‘Sociodemographic + CS/SCS’ (Step 2)……………………………………………………………..26

**Table S21**: Hierarchical multiple regression analysis predicting SWEMWBS with ‘Sociodemographic + CS/SCS’ (Step 2)……………………………………………………………..27

**Table S22**: Descriptive data and Pearson’s raw correlations between self-compassion and compassion for others per psychological distress and wellbeing subgroups and differences between groups………………………………………………………………………………………………...28

**References** (Supplements)………………………………………………………………………...29

**Table S1:** *Sociodemographic characteristics of the retest sample (n = 288)*

| Gender (women): n (%) | 232 (80.6) |
| --- | --- |
| Age (in years): M (SD) | 45.31 (11.99) |
| Marital status: n (%) |  |
| Single | 91 (31.6) |
| Married/civil partner | 158 (54.9) |
| Separated/divorced | 32 (11.1) |
| Widowed | 7 (2.4) |
| Children: n (%) |  |
| 0 | 139 (48.3) |
| 1 | 46 (16) |
| 2 | 88 (30.6) |
| 3 | 12 (4.2) |
| 4 | 3 (1) |
| Level of education: n (%) |  |
| No schooling | 1 (0.3) |
| Primary school | 10 (3.5) |
| Secondary school | 29 (10.1) |
| University | 248 (86.1) |
| Work status: n (%) |  |
| Student | 27 (9.4) |
| Unemployed | 18 (6.3) |
| Employed | 203 (70.49) |
| Self-employed | 12 (4.17) |
| Homemaker | 5 (1.7) |
| On a sick leave | 6 (2.08) |
| Retired/pensioner | 14 (4.86) |
| Unable to work | 3 (1) |

*Note.* M = mean. SD = standard deviation. n = frequencies. % = percentages.


**Table S2:** *Sociodemographic data of the non-completers Spanish native speakers (n = 351)*

| Gender (women): n (%) | 168 (47.9) |
| --- | --- |
| Age (in years): M (SD) | 39.18 (13.13) |
| Marital status: n (%) |  |
| Single | 144 (41) |
| Married/civil partner | 179 (51) |
| Separated/divorced | 25 (7.1) |
| Widowed | 3 (0.9) |
| Children: n (%) |  |
| 0 | 18 (5.1) |
| 1 | 5 (1.4) |
| 2 | 6 (1.7) |
| 3 | 1 (0,3) |
| ≥ 4 | 2 (0.6) |
| N/A | 319 |
| Level of education: n (%) |  |
| No schooling | 1 (0.3) |
| Primary school | 12 (3.4) |
| Secondary school | 98 (27.9) |
| University | 240 (68.4) |
| Work status: n (%) |  |
| Student | 70 (20) |
| Unemployed | 17 (4.8) |
| Employed | 252 (71.8) |
| Self-employed | 5 (1.5) |
| Homemaker | 2 (0.6) |
| Retired/pensioner | 5 (1.4) |

*Note.* M = mean. SD = standard deviation. n = frequencies. % = percentages.

**Figure S1:** *Bifactor exploratory measurement models*

| 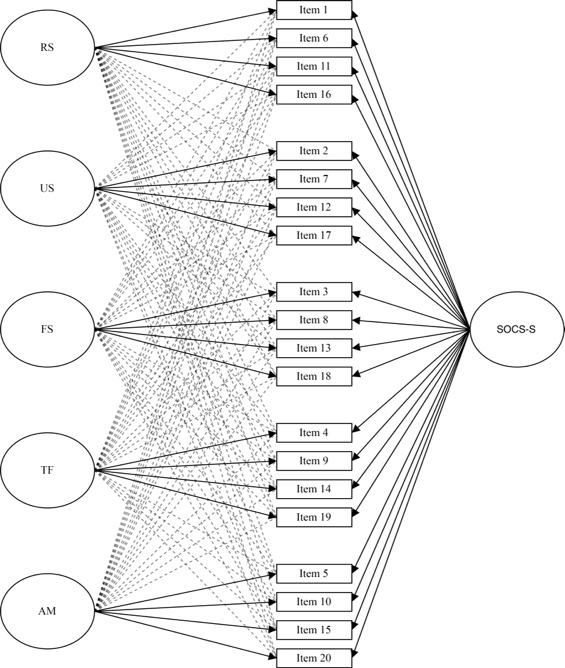 | 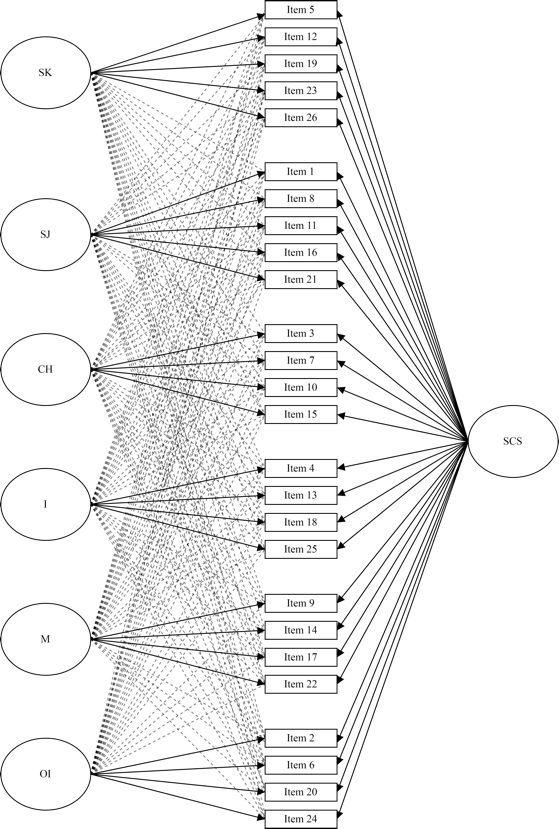 |
| --- | --- |
| Sussex-Oxford Compassion Scale-Self (SOCS-S) | Self-Compassion Scale (SCS) |
| 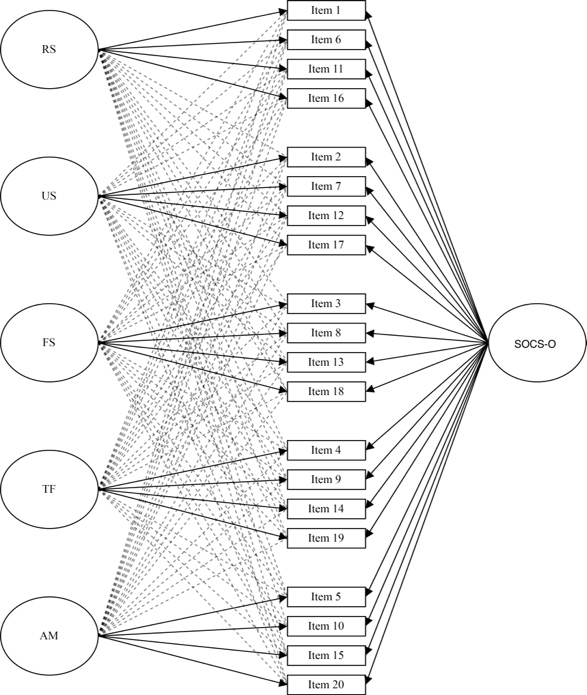 | 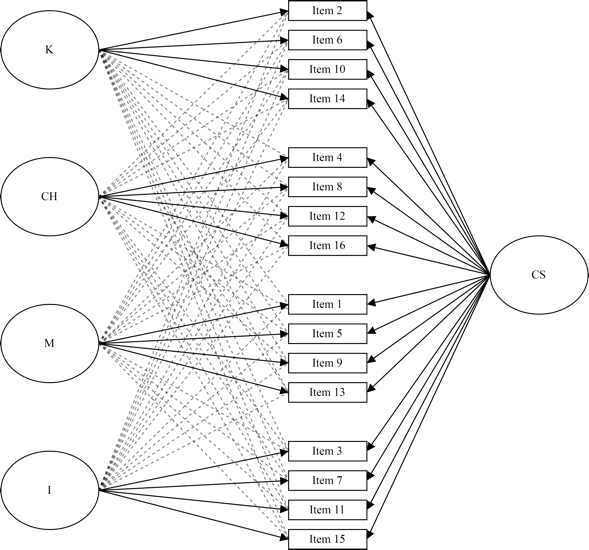 |
| Sussex-Oxford Compassion Scale-Others (SOCS-O) | Compassion Scale (CS) |

**Table S3:** *Statistical procedures used to address the construct validity, internal consistency, and test-retest validity of the measurement models for the compassion scales*

The distribution of the SOCS-S, SOCS-O, SCS, and CS items was described using means (standard deviations), skewness and kurtosis. Corrected item-total correlations also were computed, as were factor loadings on the general and specific factors.

The factor structure of the SOCS-S, SOCS-O, SCS, and CS was examined using exploratory structural equation modelling (ESEM), and confirmatory factor analysis (CFA), with the weighted least squares mean- and variance-adjusted estimator (WLSMV). The total sample (n = 811) was randomly split into two subsamples (calibration and validation subsamples) in which ESEM (n = 402) and CFA (n = 409) were conducted, respectively. In CFA, items load on the specified target factor exclusively, and cross-loadings are constrained to zero, which is a strong assumption. In contrast, ESEM allows items to load on nontarget factors as well, although targeting those cross-loadings to be as close to zero as possible (Morin et al., 2020).

We reproduced all the factor models that were originally evaluated in Gu et al. (2020) and Neff (2003) and added the bifactor structure to obtain and evaluate a more informative representation of the one-dimensionality of the general factors, in a similar way than the Neff’s models have been recently proposed and evaluated (Neff et al., 2019; Pommier, Neff & Tóth-Király, 2020). Thus, four corresponding ESEM and CFA models were tested for the SOCS-S and SOCS-O: i) a one-factor model with a single self-compassion/compassion for others factor; ii) a five-factor correlated model with five factors representing the subscales; iii) a five-factor hierarchical model in which the factors load on an overarching compassion factor; and iv) a bifactor model with a general compassion factor and the five orthogonal factors. Four corresponding ESEM and CFA models were also tested for the SCS: i) a one-factor model with a single self-compassion factor; ii) a six-factor correlated model with six factors representing the subscales; iii) a single-hierarchical model in which the six factors load on an overarching self-compassion factor; and iv) a bifactor model with a general self-compassion factor and the six orthogonal factors. Finally, four corresponding ESEM and CFA models were tested for the CS: i) a one-factor model with a single compassion factor; ii) a four-factor correlated model with four factors representing the subscales; iii) a hierarchical model in which the four factors load on an overarching compassion factor; and iv) a bifactor model with a general compassion factor and the four orthogonal factors. Before those analyses, SCS and CS negative items were reverse-coded. In the model–data fit assessment, the following goodness-of-fit indices were examined with their respective cut-off points (Hu & Bentler, 1999; Schermelleh-Engel et al., 2003): the comparative fit index (CFI; ≥ .95 for good, ≥ .90 for acceptable), the Tucker–Lewis index (TLI; ≥ .95 for good, ≥ .90 for acceptable), the root-mean-square error of approximation (RMSEA; ≤ .06 for good, ≤ .10 for acceptable) with its 90% confidence interval, and the weighted root-mean-square residual (WRMR; ≤ 1.00 for acceptable).

The internal consistency of the scales was determined by calculating Cronbach’s alpha (α) and McDonald’s omega (ω), with coefficients ≥ .70 indicating adequate internal consistency (DeVellis, 1991). McDonald’s ω has the advantage of considering the strength of association between items and constructs as well as item-specific measurement errors, providing a more realistic estimate of true reliability (McDonald, 1999). For the ESEM bifactor models, omega hierarchical (ω_h_), construct replicability (coefficient H), explained common variance (ECV), percent of uncontaminated correlations (PUC), and the factor determinacy index (FDI) were calculated. Omega hierarchical reflects the proportion of variance that can be attributed to a factor after removing the variance explained by the rest of the factors of the scale (Rodriguez et al., 2016). In the case of general factors, an ω_h_ of approximately .80 suggests that the scale can be considered unidimensional (Reise et al., 2013), and regarding the specific factors, low ω_h_ values (< .50) indicate that the computation of specific subscale scores is not recommended (Brunner et al., 2012). Construct replicability (H) indicates how well latent variables are defined by evaluating the proportion of the factor variance that can be accounted for by its indicators, with H ≥ .70 indicating a reasonable definition and H ≥ .80 suggesting an adequately defined construct (Hancock & Mueller, 2000; Rodriguez et al., 2016). ECV represents the proportion of common variance attributable to the general factor (for specific factors, ECV shows the strength of the factor relative to the variance that all items in the scale explain), and PUC is the number of correlations between items from different factors divided by the total number of correlations, which indicates the proportion of correlations reflecting the possible general factor. Values ≥ .70 for both indices reflect that the common variance of a scale can be considered unidimensional, and this is also true if the PUC is < .80 but the ECV is > .60 and the ω_H_ is > .70 (Rodriguez et al., 2016). FDI is the correlation between the factor score estimates and the levels on the latent factors they estimate (Beauducel, 2011), with FDI values around .80 indicating that factor score estimates are adequate, and FDI ≥ .90 are high (Gorsuch, 1983).

Test-retest reliability at one week (for the SOCS-S and SOCS-O only) was assessed using the intraclass correlation coefficient (ICC). The ICC estimates were calculated based on mean measurements, absolute agreement and a 2-way mixed-effects model. The results were interpreted in accordance with the following guidelines (Koo & Li, 2016): < .50 for poor test-retest reliability, between .50 and .75 for moderate test-retest reliability, between .75 and .90 for good test-retest reliability, and > .90 for excellent.

**Table S4:** *Spanish adaptation process of the SOCS-O, SOCS-S, and CS*

The translation of the SOCS-O, SOCS-S, and CS scales into Spanish was carried out following the usual procedures of cross-cultural scale translation (Guillemin et al., 1993). Firstly, the scale was translated from English into Spanish according to the parallel back-translation procedure (Brislin, 1986), in which the scale is initially translated from the original language into the language of the study by two independent bilingual subjects. Two bilingual researchers, unaware of the objectives of the scales, provided the initial Spanish translation, and each researcher translated the scales separately. This translation was then back translated to the original English language by two other bilingual linguistic experts who did not have specific knowledge of the instruments. This complete sequence involved four bilingual subjects carrying out the parallel back-translation procedure to ensure correct translation and avoid possible biases, thus obtaining two pilot versions in Spanish of these scales. Secondly, the items obtained were evaluated by a committee composed of the subjects who participated in the translation process and two expert researchers in Compassion Psychology, who selected the items that had maintained the original meaning and prepared the format and instructions of the scale in an identical way to the original English version. Any discrepancies were solved through mutual agreement, and both the translators and researchers were present during the agreement. An independent native English-speaking teacher determined the equivalence of the two English versions of this assessment, the original and the one that resulted from this process. Finally, to determine clarity in the formulation of items, the corresponding Spanish version of the scale was administered to 12 university students who provided comments on the instructions and items, from which few modifications were made.

**Table S5:** *Sussex-Oxford Compassion for Others Scale (SOCS-O) Spanish version*

| Debajo hay frases que describen cómo podríamos relacionarnos con otras personas. Por favor, indique hasta qué punto son ciertas para usted usando la siguiente escala de respuestas de 5 puntos (1 = No es verdad en absoluto, 2 = Raramente es verdad, 3 = A veces es verdad, 4 = Casi siempre es verdad, 5 = Siempre es verdad). Por ejemplo, si piensa que la frase casi siempre es verdad, dibuje un círculo en el “4”. En los ítems de debajo, las palabras generales (ej: ‘disgustado’, ‘angustiado’, ‘sufrimiento’, ‘apuros’) se utilizan para cubrir un rango de emociones desagradables como tristeza, miedo, ira, frustración, culpa, vergüenza, etc.  Por favor, proporcione una respuesta a cada frase.  1. Me doy cuenta cuando otras personas se sienten angustiadas sin que tengan que decírmelo.  2. Entiendo que todas las personas experimentan sufrimiento en algún momento de sus vidas.  3. Cuando alguien atraviesa un momento difícil, siento compasión hacia esa persona.  4. Cuando alguien está disgustado, trato de permanecer abierto a sus sentimientos en lugar de evitarlos.  5. Cuando otros pasan apuros, trato de hacer cosas que puedan ayudarles.  6. Me doy cuenta cuando otros se sienten angustiados.  7. Entiendo que sentirse disgustado a veces es parte de la naturaleza humana.  8. Cuando escucho que a otras personas les ocurren cosas negativas, me preocupo por su bienestar.  9. Permanezco al lado y escucho a otras personas cuando están disgustadas, incluso si es difícil de soportar.  10. Cuando alguien atraviesa un momento complicado, trato de cuidarlo.  11. Noto rápidamente los primeros signos de angustia en los demás.  12. Al igual que yo, sé que otras personas también pasan apuros en la vida.  13. Cuando alguien está disgustado, trato de sintonizar con cómo se siente.  14. Conecto con el sufrimiento de los demás sin juzgarlos.  15. Cuando veo que alguien está necesitado, intento hacer lo que es mejor para él.  16. Reconozco los signos de sufrimiento en los demás.  17. Se que todos podemos sentirnos mal a veces cuando sufrimos una injusticia.  18. Soy sensible a la angustia de los demás.  19. Cuando alguien está disgustado, puede contar conmigo sin que yo esté abrumado por su malestar.  20. Cuando veo que alguien está mal, hago todo lo posible para cuidarlo. |
| --- |

**Table S6:** *Sussex-Oxford Compassion for the Self Scale (SOCS-S) Spanish version*

| Debajo hay frases que describen cómo usted podría relacionarse consigo mismo. Por favor, indique hasta qué punto son ciertas para usted usando la siguiente escala de respuestas de 5 puntos (1 = No es verdad en absoluto, 2 = Raramente es verdad, 3 = A veces es verdad, 4 = Casi siempre es verdad, 5 = Siempre es verdad). Por ejemplo, si piensa que la frase casi siempre es verdad, dibuje un círculo en el “4”. En los ítems de debajo, las palabras generales (ej: ‘disgustado’, ‘angustiado’, ‘sufrimiento’, ‘apuros’) se utilizan para cubrir un rango de emociones desagradables como tristeza, miedo, ira, frustración, culpa, vergüenza, etc.  Por favor, proporcione una respuesta a cada frase.  1. Reconozco perfectamente cuando me siento angustiado.  2. Entiendo que todos experimentamos sufrimiento en algún momento de nuestras vidas.  3. Cuando estoy atravesando por un momento difícil, siento compasión hacia mí mismo.  4. Cuando estoy disgustado, intento estar abierto a mis sentimientos en lugar de evitarlos.  5. Intento animarme cuando estoy angustiado, incluso si es imposible actuar sobre la causa  6. Noto cuando estoy angustiado  7. Entiendo que sentirse disgustado a veces es parte de la naturaleza humana.  8. Cuando me ocurren cosas negativas, cuido de mí mismo.  9. Conecto con mi propio malestar sin dejar que me sobrepase.  10. Cuando paso por momentos difíciles, intento cuidar de mí mismo.  11. Noto rápidamente los primeros signos de angustia en mí mismo.  12. Al igual que yo, se que otras personas también pasan apuros en la vida.  13. Cuando estoy disgustado, intento sintonizar con mis sentimientos  14. Conecto con mi propio sufrimiento sin juzgarme a mí mismo  15. Cuando estoy disgustado, intento hacer lo que es mejor para mí.  16. Reconozco los signos de sufrimiento en mí mismo.  17. Se que todos podemos sentirnos angustiados cuando las cosas no van bien en nuestras vidas.  18. Incluso cuando estoy decepcionado conmigo mismo, puedo darme afecto cuando estoy angustiado  19. Cuando estoy disgustado, puedo aceptar la presencia de mis emociones sin sentirme sobrepasado.  20. Cuando estoy disgustado, hago todo lo posible para cuidar de mí mismo. |
| --- |

**Table S7:** *Descriptive statistics of the SOCS-O and standardised factor loadings for the bifactor exploratory measurement model (n = 811)*

|  |  |  |  |  |  |  | **λ spec** | | | | |
| --- | --- | --- | --- | --- | --- | --- | --- | --- | --- | --- | --- |
| **Items** |  | ***M (SD)*** | **S** | **K** | ***r_tot_*** | **λ gen** | **I** | **II** | **III** | **IV** | **V** |
| **Factor I. Recognising suffering** |  |  |  |  |  |  |  |  |  |  |  |
| 1. I recognise when other people are feeling distressed without them having to tell me. |  | 3.91 (0.63) | -.34 | .56 | .73 | .48 | **.74** | .06 | -.08 | -.06 | -.08 |
| 6. I notice when others are feeling distressed. |  | 4.03 (0.68) | -.40 | .45 | .78 | .53 | **.75** | .08 | -.09 | -.06 | -.08 |
| 11. I’m quick to notice early signs of distress in others. |  | 3.76 (0.77) | -.31 | .15 | .74 | .48 | **.72** | .01 | *.04* | .06 | .05 |
| 16. I recognise signs of suffering in others. |  | 3.94 (0.69) | -.22 | -.16 | .76 | .53 | **.71** | .12 | .18 | .13 | *.02* |
| **Factor II. Understanding the universality of suffering** |  |  |  |  |  |  |  |  |  |  |  |
| 2. I understand that everyone experiences suffering at some point in their lives. |  | 4.61 (0.61) | -1.61 | 3.26 | .72 | .40 | .10 | **.78** | *.02* | *-.04* | -.07 |
| 7. I understand that feeling upset at times is part of human nature. |  | 4.48 (0.73) | -1.41 | 1.93 | .69 | .41 | .04 | **.76** | -.11 | *-.06* | *.00* |
| 12. Like me, I know that other people also experience struggles in life. |  | 4.58 (0.63) | -1.58 | 3.22 | .74 | .44 | .10 | **.77** | *.02* | .06 | *-.06* |
| 17. I know that we can all feel upset at times when we are wronged. |  | 4.60 (0.59) | -1.31 | 1.7 | .61 | .48 | .03 | **.63** | .19 | *.06* | *.02* |
| **Factor III. Feeling for the person suffering** |  |  |  |  |  |  |  |  |  |  |  |
| 3. When someone is going through a difficult time, I feel kindly towards them. |  | 4.28 (0.70) | -.79 | .73 | .54 | .66 | -.09 | .12 | **.33** | -.09 | -.11 |
| 8. When I hear about bad things happening to other people, I feel concern for their wellbeing. |  | 4.11 (0.72) | -.41 | -.09 | .63 | .83 | -.07 | *-.03* | **.09** | *-.05* | .11 |
| 13. When someone is upset, I try to tune in to how they’re feeling. |  | 4.03 (0.74) | -.50 | .36 | .61 | .79 | .10 | *-.03* | **.13** | .12 | .06 |
| 18. I’m sensitive to other people’s distress. |  | 4.19 (0.75) | -.83 | 1 | .55 | .64 | .16 | .11 | **.32** | *-.01* | .09 |
| **Factor IV. Tolerating uncomfortable feelings** |  |  |  |  |  |  |  |  |  |  |  |
| 4. When someone else is upset, I try to stay open to their feelings rather than avoid them. |  | 4.05 (0.71) | .71 | -.14 | .63 | .82 | -.07 | *-.04* | *.00* | **.18** | -.24 |
| 9. I stay with and listen to other people when they’re upset even if it’s hard to bear. |  | 4.02 (9.73) | .73 | -.23 | .65 | .78 | .02 | *-.00* | -.17 | **.25** | *.05* |
| 14. I connect with the suffering of others without judging them. |  | 3.77 (0.77) | .77 | -.12 | .62 | .67 | .10 | *.01* | .13 | **.41** | *.02* |
| 19. When someone else is upset, I can be there for them without feeling overwhelmed by their distress. |  | 4.01 (0.75) | .75 | -.43 | .60 | .65 | .00 | *.05* | -.10 | **.34** | .14 |
| **Factor V. Acting or being motivated to act to alleviate suffering** |  |  |  |  |  |  |  |  |  |  |  |
| 5. When others are struggling, I try to do things that would be helpful. |  | 4.16 (0.66) | -.31 | -.19 | .71 | .84 | -.10 | -.05 | .07 | -.13 | **.16** |
| 10. When someone is going through a difficult time, I try to look after them. |  | 4.15 (0.68) | -.34 | -.31 | .78 | .89 | -.08 | -.11 | -.09 | *-.05* | **.23** |
| 15. When I see someone in need, I try to do what’s best for them. |  | 3.97 (0.72) | -.27 | -.26 | .70 | .72 | -.04 | *-.04* | .07 | .09 | **.40** |
| 20. When I see that someone is upset, I do my best to take care of them. |  | 4.04 (0.73) | -.25 | -.58 | .80 | .84 | -.03 | -.08 | *-.01* | .06 | **.43** |

*Note.* M = mean; SD = standard deviation; S = skewness; K = kurtosis. r_tot_ = corrected item-total correlations. Standardised factor loadings on the general compassion factor (λ gen) and specific factors (λ spec). Target factor loadings are in bold. Nonsignificant parameters (*p* ≥.05) are italicised.

**Table S8:** *Descriptive statistics of the SOCS-S and standardised factor loadings for the bifactor exploratory measurement model (n = 811)*

|  |  |  |  |  |  |  | **λ spec** | | | | |
| --- | --- | --- | --- | --- | --- | --- | --- | --- | --- | --- | --- |
| **Items** |  | ***M (SD)*** | **S** | **K** | ***r_tot_*** | **λ gen** | **I** | **II** | **III** | **IV** | **V** |
| **Factor I. Recognising suffering** |  |  |  |  |  |  |  |  |  |  |  |
| 1. I’m good at recognising when I’m feeling distressed. |  | 4.22 (0.65) | -.38 | -.11 | .69 | .38 | **.75** | .16 | .21 | *-.04* | *-.03* |
| 6. I notice when I’m feeling distressed. |  | 4.28 (0.68) | -.64 | .43 | .74 | .40 | **.81** | .07 | .08 | -.07 | -.11 |
| 11. I’m quick to notice early signs of distress in myself. |  | 3.91 (0.87) | -.62 | .15 | .70 | .40 | **.71** | *.03* | -.13 | *.04* | .09 |
| 16. I recognise signs of suffering in myself. |  | 4.07 (0.79) | -.67 | .53 | .72 | .49 | **.70** | .16 | -.22 | *.03* | *.05* |
| **Factor II. Understanding the universality of suffering** |  |  |  |  |  |  |  |  |  |  |  |
| 2. I understand that everyone experiences suffering at some point in their lives. |  | 4.59 (0.64) | -1.57 | 2.65 | .69 | .36 | .10 | **.79** | .24 | *-.05* | *-.07* |
| 7. I understand that feeling upset at times is part of human nature. |  | 4.34 (0.84) | -1.26 | 1.22 | .66 | .47 | *.05* | **.66** | *.01* | *-.05* | -.06 |
| 12. Like me, I know that other people also experience struggles in life. |  | 4.56 (0.67) | -1.55 | 2.37 | .74 | .42 | .09 | **.79** | -.07 | *.02* | *.01* |
| 17. I know that we can all feel upset at times when we are wronged. |  | 4.52 (0.69) | -1.39 | 1.81 | .71 | .38 | .15 | **.79** | -.20 | *.07* | *.06* |
| **Factor III. Feeling for the person suffering** |  |  |  |  |  |  |  |  |  |  |  |
| 3. When I’m going through a difficult time, I feel kindly towards myself. |  | 3.18 (0.98) | -.07 | -.44 | .51 | .56 | *.03* | *.02* | ***.11*** | -.14 | *-.03* |
| 8. When bad things happen to me, I feel caring towards myself. |  | 3.45 (0.95) | -.23 | -.38 | .71 | .86 | -.04 | -.07 | ***.06*** | -.09 | .33 |
| 13. When I'm upset, I try to tune in to how I'm feeling. |  | 3.60 (0.90) | -.29 | -.42 | .67 | .86 | .10 | *.04* | **-.14** | *-.01* | -.10 |
| 18. Even when I'm disappointed with myself, I can feel warmly towards myself when I'm in distress. |  | 3.25 (0.99) | -.14 | -.55 | .70 | .83 | *-.04* | *-.04* | **.19** | .13 | .09 |
| **Factor IV. Tolerating uncomfortable feelings** |  |  |  |  |  |  |  |  |  |  |  |
| 4. When I’m upset, I try to stay open to my feelings rather than avoid them. |  | 3.55 (0.93) | -.26 | -.53 | .61 | .75 | .06 | *.04* | -.09 | ***-.04*** | -.29 |
| 9. I connect with my own distress without letting it overwhelm me. |  | 3.24 (0.95) | -.17 | -.43 | .71 | .79 | -.08 | *.04* | *-.05* | **.20** | *.01* |
| 14. I connect with my own suffering without judging myself. |  | 3.04 (0.99) | -.07 | -.47 | .73 | .82 | *-.02* | *-.04* | *.03* | **.15** | *-.03* |
| 19. When I’m upset, I can let the emotions be there without feeling overwhelmed. |  | 3.26 (0.95) | -.21 | -.37 | .75 | .81 | *-.03* | -.01 | *.02* | **.52** | *.01* |
| **Factor V. Acting or being motivated to act to alleviate suffering** |  |  |  |  |  |  |  |  |  |  |  |
| 5. I try to make myself feel better when I’m distressed, even if I can’t do anything about the cause. |  | 3.54 (0.87) | -.17 | -.39 | .58 | .70 | -.11 | *-.03* | *.01* | *.01* | **-.09** |
| 10. When I’m going through a difficult time, I try to look after myself. |  | 3.45 (0.96) | -.18 | -.45 | .78 | .88 | -.05 | *-.02* | *.04* | -.07 | **.38** |
| 15. When I’m upset, I try to do what’s best for myself. |  | 3.45 (0.90) | -.22 | -.19 | .72 | .79 | *-.01* | *-.03* | *-.01* | .10 | **.19** |
| 20. When I’m upset, I do my best to take care of myself. |  | 3.33 (0.98) | -.12 | -.44 | .80 | .85 | *-.01* | -.10 | .11 | .07 | **.31** |

*Note.* M = mean; SD = standard deviation; S = skewness; K = kurtosis. rtot = corrected item-total correlations. Standardised factor loadings on the general self-compassion factor (λ gen) and specific factors (λ spec). Target factor loadings are in bold. Nonsignificant parameters (p ≥.05) are italicised.

**Table S9:** *Reliability indices of the bifactor exploratory measurement model for the Sussex-Oxford Compassion Scales (SOCS-S and SOCS-O)*

|  | **Compassion for Others (SOCS-O)** | | | | | | | | | | | | | | |  | **Compassion for the Self (SOCS-S)** | | | | | | | | | | | | | | |
| --- | --- | --- | --- | --- | --- | --- | --- | --- | --- | --- | --- | --- | --- | --- | --- | --- | --- | --- | --- | --- | --- | --- | --- | --- | --- | --- | --- | --- | --- | --- | --- |
| **Scale** | **Test**  (*n* = 811) | | | | | |  | **Retest**  (*n* = 288) | | | | | |  |  |  | **Test**  (*n* = 811) | | | | | |  | **Retest**  (*n* = 283) | | | | | |  |  |
|  | **α** | **ω** | **ω_h_** | **H** | **ECV** | **FDI** |  | **α** | **ω** | **ω_h_** | **H** | **ECV** | **FDI** |  | **ICC** |  | **α** | **ω** | **ω_h_** | **H** | **ECV** | **FDI** |  | **α** | **ω** | **ω_h_** | **H** | **ECV** | **FDI** |  | **ICC** |
| GF | .92 | .97 | .86 | .96 | .62 | .97 |  | .92 | .97 | .85 | .95 | .58 | .96 |  | .86 |  | .93 | .97 | .87 | .96 | .63 | .98 |  | .94 | .98 | .89 | .97 | .65 | .98 |  | .88 |
| RS | .91 | .94 | .63 | .82 | .15 | .94 |  | .91 | .97 | .69 | .87 | .17 | .96 |  | .81 |  | .86 | .91 | .70 | .84 | .16 | .94 |  | .89 | .95 | .65 | .84 | .14 | .96 |  | .75 |
| US | .86 | .92 | .68 | .84 | .15 | .94 |  | .84 | .92 | .71 | .85 | .16 | .94 |  | .72 |  | .85 | .92 | .71 | .85 | .16 | .95 |  | .93 | .97 | .68 | .87 | .16 | .97 |  | .73 |
| FS | .79 | .85 | .07 | .21 | .02 | .54 |  | .79 | .87 | .15 | .42 | .04 | .75 |  | .81 |  | .85 | .87 | .00 | .07 | .01 | .44 |  | .83 | .90 | .02 | .20 | .01 | .74 |  | .85 |
| TF | .83 | .87 | .12 | .30 | .03 | .65 |  | .80 | .87 | .11 | .34 | .03 | .75 |  | .81 |  | .88 | .90 | .06 | .30 | .02 | .84 |  | .88 | .94 | .08 | .33 | .03 | .83 |  | .86 |
| AM | .88 | .94 | .11 | .33 | .03 | .74 |  | .87 | .93 | .14 | .36 | .03 | .73 |  | .84 |  | .89 | .91 | .05 | .25 | .02 | .76 |  | .89 | .92 | .06 | .19 | .01 | .67 |  | .87 |

*Note.* GF = general factor; RS = recognising suffering; US = understanding the universality of suffering; FS = feeling for the person suffering; TF = tolerating uncomfortable feelings; AM = acting or being motivated to act to alleviate suffering; α = Cronbach’s alpha; ω = omega composite reliability; ω_h_ = omega hierarchical; H = construct replicability; ECV = explained common variance; FDI = factor determinacy index; ICC = intraclass correlation coefficient (test/retest reliability). The percent of uncontaminated correlations (PUC) in all the models was PUC = .84.

**Table S10:** *Goodness-of-fit indices of factor models for the Sussex-Oxford Compassion Scales (SOCS-S and SOCS-O)*

|  |  | **Exploratory Structural Equation Modelling (ESEM)** | | | |  | **Confirmatory Factor Analysis (CFA)** | | | |
| --- | --- | --- | --- | --- | --- | --- | --- | --- | --- | --- |
| **Scale** | **Model** | **CFI** | **TLI** | **RMSEA [90%CI]** | **WRMR** |  | **CFI** | **TLI** | **RMSEA [90%CI]** | **WRMR** |
|  | One-factor | .857 | .840 | .200 [.194, .207] | 3.573 |  | .824 | .803 | .209 [.203, .216] | 3.786 |
| Compassion | Five-factor | .992 | .985 | .062 [.052, .071] | 0.521 |  | .912 | .896 | .152 [.145, .159] | 2.426 |
| for the Self | Hierarchical five-factor | .991 | .983 | .065 [.056, .074] | 0.607 |  | .975 | .971 | .080 [.073, .087] | 1.425 |
|  | Bifactor | .995 | .988 | .054 [.043, .065] | 0.418 |  | .976 | .970 | .082 [.075, .090] | 1.322 |
|  | One-factor | .839 | .820 | .179 [.173, .186] | 3.274 |  | .809 | .787 | .202 [.196, .209] | 3.823 |
| Compassion | Five-factor | .991 | .983 | .054 [.045, .064] | 0.500 |  | .984 | .981 | .061 [.054, .069] | 1.028 |
| for Others | Hierarchical five-factor | .991 | .984 | .054 [.045, .064] | 0.544 |  | .981 | .978 | .065 [.058, .072] | 1.182 |
|  | Bifactor | .995 | .989 | .044 [.032, .055] | 0.386 |  | .982 | .977 | .067 [.059, .074] | 1.084 |

*Note.* ESEM sample = 402, CFA sample = 409. CFI = comparative fit index; TLI = Tucker‒Lewis index; RMSEA = root mean square error approximation; 90% CI = 90% confidence interval of the RMSEA; WRMR = weighted root means square residual. The chosen estimator was diagonally weighted least squares (WLSMV). When the five-factor hierarchical models of the SOCS-O and SOCS-S were examined with CFA, a Heywood case appeared in each analysis. Specifically, the relationship between the overarching compassion factor and ‘Feeling’ showed a standardized coefficient greater than one and a negative residual variance in both cases. Following Dillon et al. (1987) recommendations, this residual variance was fixed to 0 and then the model perfectly converged. In addition, the correlated five-factor CFA model of the SOCS-S showed a Heywood case. It was due to a correlation greater than one between the factors ‘Acting’ and ‘Feeling’, which was solved by fixing the residual variance of ‘Feeling’ to 1.

**Table S11:** *Self-Compassion Scale (SCS) Spanish version* (García-Campayo et al., 2014)

¿CÓMO ACTÚO HABITUALMENTE HACIA MÍ MISMO EN MOMENTOS DIFÍCILES?

Lea cada frase cuidadosamente antes de contestar. A la izquierda de cada frase, indique la frecuencia con que se comporta de la manera indicada, utilizando la siguiente escala: 1 (casi nunca), 2, 3, 4, 5 (casi siempre)

1. Desapruebo mis propios defectos e incapacidades y soy crítico/a respecto a ellos.

2. Cuando me siento bajo/a de ánimo, tiendo a obsesionarme y a fijarme en todo lo que va mal.

3. Cuando las cosas me van mal, veo las dificultades como parte de lo que a todo el mundo le toca vivir

4. Cuando pienso en mis deficiencias, tiendo a sentirme más separado/a y aislado/a del resto del mundo.

5. Trato de ser cariñoso/a conmigo mismo/a cuando siento malestar emocional.

6. Cuando fallo en algo importante para mí, me consumen los sentimientos de ineficacia.

7. Cuando estoy desanimado y triste, me acuerdo de que hay muchas personas en el mundo que se sienten como yo.

8. Cuando vienen épocas muy difíciles, tiendo a ser duro/a conmigo mismo/a.

9. Cuando algo me disgusta trato de mantener mis emociones en equilibrio.

10. Cuando me siento incapaz de alguna manera, trato de recordarme que casi todas las personas comparten sentimientos de incapacidad.

11. Soy intolerante e impaciente con aquellos aspectos de mi personalidad que no me gustan.

12. Cuando lo estoy pasando verdaderamente mal, me doy el cuidado y el cariño que necesito.

13. Cuando estoy bajo/a de ánimo, tiendo a pensar que, probablemente, la mayoría de la gente es más feliz que yo.

14. Cuando me sucede algo doloroso trato de mantener una visión equilibrada de la situación.

15. Trato de ver mis defectos como parte de la condición humana.

16. Cuando veo aspectos de mí mismo/a que no me gustan, me critico continuamente.

17. Cuando fallo en algo importante para mí, trato de ver las cosas con perspectiva.

18. Cuando realmente estoy en apuros, tiendo a pensar que otras personas lo tienen más fácil.

19. Soy amable conmigo mismo/a cuando estoy experimentando sufrimiento.

20. Cuando algo me molesta me dejo llevar por mis sentimientos.

21. Puedo ser un poco insensible hacia mí mismo/a cuando estoy experimentando sufrimiento.

22. Cuando me siento deprimido/a trato de observar mis sentimientos con curiosidad y apertura de mente.

23. Soy tolerante con mis propios defectos e imperfecciones o debilidades.

24. Cuando sucede algo doloroso tiendo a hacer una montaña de un grano de arena.

25. Cuando fallo en algo que es importante para mí, tiendo a sentirme solo en mi fracaso.

26. Trato de ser comprensivo y paciente con aquellos aspectos de mi personalidad que no me gustan.

**Table S12:** *Descriptive statistics of the SCS and standardised factor loadings for the bifactor exploratory measurement model (n = 811)*

|  |  |  |  |  |  | **λ spec** | | | | | |
| --- | --- | --- | --- | --- | --- | --- | --- | --- | --- | --- | --- |
| **Items** | ***M (SD)*** | **S** | **K** | ***r_tot_*** | **λ gen** | **I** | **II** | **III** | **IV** | **V** | **VI** |
| **Factor I. Self-Kindness** |  |  |  |  |  |  |  |  |  |  |  |
| 5. I try to be loving towards myself when I’m feeling emotional pain. | 3.29 (1.18) | -.21 | -.80 | .75 | .74 | **.35** | *.04* | .10 | -.09 | .08 | -.12 |
| 12. When I’m going through a very hard time, I give myself the caring and tenderness I need. | 3.04 (1.16) | -.05 | -.80 | .74 | .74 | **.42** | *-.05* | .09 | -.13 | .08 | -.07 |
| 19. I’m kind to myself when I’m experiencing suffering. | 3.20 (1.13) | -.14 | -.69 | .80 | .81 | **.37** | *-.03* | .04 | -.08 | .10 | -.11 |
| 23. I’m tolerant of my own flaws and inadequacies. | 3.15 (1.17) | -.13 | -.84 | .68 | .81 | **-.14** | .08 | *-.03* | -.09 | .*04* | -.29 |
| 26. I try to be understanding and patient towards those aspects of my personality I don't like. | 3.23 (1.12) | -.15 | -.67 | .77 | .85 | ***-.04*** | .06 | *.00* | -.08 | .11 | -.21 |
| **Factor II. Self-Judgement** |  |  |  |  |  |  |  |  |  |  |  |
| 1. I’m disapproving and judgmental about my own flaws and inadequacies. | 2.73 (1.25) | .26 | -.87 | .63 | .65 | -.12 | **.33** | -.*05* | -.06 | -.17 | .*01* |
| 8. When times are really difficult, I tend to be tough on myself | 3.07 (1.25) | -.04 | -1.02 | .73 | .74 | .09 | **.30** | -.06 | .09 | -.12 | .14 |
| 11. I’m intolerant and impatient towards those aspects of my personality I don't like. | 3.22 (1.23) | -.16 | -.98 | .72 | .73 | -.08 | **.39** | -.09 | -.*01* | -.*02* | .06 |
| 16. When I see aspects of myself that I don’t like, I get down on myself. | 3.22 (1.26) | -.16 | -1.00 | .74 | .77 | -.08 | **.30** | -.10 | .*04* | -.11 | .09 |
| 21. I can be a bit cold-hearted towards myself when I'm experiencing suffering. | 3.26 (1.20) | -.13 | -.95 | .64 | .72 | .18 | **.21** | .*00* | .12 | -.*01* | .08 |
| **Factor III. Common humanity** |  |  |  |  |  |  |  |  |  |  |  |
| 3. When things are going badly for me, I see the difficulties as part of life that everyone goes through. | 3.40 (1.15) | -.28 | -.80 | .53 | .33 | *.04* | *-.05* | **.53** | .*02* | .16 | -.07 |
| 7. When I'm down, I remind myself that there are lots of other people in the world feeling like I am. | 2.99 (1.25) | .05 | -.97 | .56 | .34 | .07 | *.01* | **.64** | .*00* | .*02* | .*01* |
| 10. When I feel inadequate in some way, I try to remind myself that feelings of inadequacy are shared by most people. | 2.91 (1.20) | .04 | -.91 | .62 | .48 | *.03* | -.14 | **.65** | -.15 | -.*02* | .06 |
| 15. I try to see my failings as part of the human condition. | 3.45 (1.18) | -.39 | -.69 | .54 | .68 | *.02* | .05 | **.35** | .*01* | .18 | -.14 |
| **Factor IV. Isolation** |  |  |  |  |  |  |  |  |  |  |  |
| 4. When I think about my inadequacies, it tends to make me feel more separate and cut off from the rest of the world. | 3.20 (1.33) | -.04 | -1.21 | .60 | .67 | -.08 | .*02* | -.08 | **.22** | -.19 | .22 |
| 13. When I’m feeling down, I tend to feel like most other people are probably happier than I am. | 3.61 (1.27) | -.53 | -.81 | .69 | .67 | -.08 | .05 | -.*03* | **.60** | .08 | .07 |
| 18. When I’m really struggling, I tend to feel like other people must be having an easier time of it. | 3.55 (1.21) | -.40 | -.87 | .55 | .44 | -.05 | .*03* | -.09 | **.61** | .10 | .*03* |
| 25. When I fail at something that's important to me, I tend to feel alone in my failure. | 3.01 (1.28) | .10 | -1.06 | .57 | .72 | *-.04* | -.06 | -.*01* | **.14** | -.23 | .22 |
| **Factor V. Mindfulness** |  |  |  |  |  |  |  |  |  |  |  |
| 9. When something upsets me I try to keep my emotions in balance. | 3.52 (1.03) | -.30 | -.44 | .64 | .61 | .11 | -.07 | .12 | -.*03* | **.44** | .11 |
| 14. When something painful happens I try to take a balanced view of the situation. | 3.53 (1.05) | -.36 | -.50 | .70 | .69 | *.01* | -.11 | .10 | .08 | **.49** | .13 |
| 17. When I fail at something important to me I try to keep things in perspective. | 3.41 (1.06) | -.30 | -.53 | .67 | .73 | .05 | -.09 | .09 | .07 | **.28** | -.*02* |
| 22. When I'm feeling down I try to approach my feelings with curiosity and openness. | 3.18 (1.15) | -.18 | -.77 | .60 | .69 | .12 | -.14 | .11 | -.08 | **.19** | -.09 |
| **Factor VI. Overidentification** |  |  |  |  |  |  |  |  |  |  |  |
| 2. When I’m feeling down I tend to obsess and fixate on everything that’s wrong. | 3.08 (1.30) | -.05 | -1.09 | .69 | .77 | *-.04* | .09 | -.*04* | .14 | .*00* | **.29** |
| 6. When I fail at something important to me I become consumed by feelings of inadequacy. | 2.68 (1.25) | .34 | -.88 | .57 | .75 | *.00* | .09 | -.10 | .*03* | -.25 | **.20** |
| 20. When something upsets me I get carried away with my feelings. | 2.91 (1.10) | .14 | -.63 | .49 | .45 | *-.02* | .20 | .*01* | .*05* | .17 | **.35** |
| 24. When something painful happens I tend to blow the incident out of proportion. | 3.36 (1.25) | -.29 | -.96 | .63 | .61 | -.10 | .*00* | -.*03* | .18 | .17 | **.45** |

*Note.* M = mean; SD = standard deviation; S = skewness; K = kurtosis. r_tot_ = corrected item-total correlations. Standardised factor loadings on the general self-compassion factor (λ gen) and specific factors (λ spec). Target factor loadings are in bold. Nonsignificant parameters (*p* ≥.05) are italicised.

**Table S13:** *Reliability indices of the bifactor exploratory measurement model for the Self-Compassion Scale (SCS)*

| **Scale** | α | ω | ω_h_ | H | ECV | FDI |
| --- | --- | --- | --- | --- | --- | --- |
| General factor | .95 | .97 | .92 | .96 | .75 | .98 |
| K | .90 | .92 | .05 | .35 | .03 | .77 |
| CH | .76 | .81 | .47 | .66 | .03 | .85 |
| MI | .83 | .85 | .18 | .40 | .08 | .74 |
| SJ | .87 | .89 | .14 | .36 | .05 | .70 |
| IS | .79 | .85 | .24 | .55 | .03 | .84 |
| OI | .78 | .82 | .16 | .34 | .03 | .68 |

*Note.* N = 811; K = kindness; CH = common humanity; MI = mindfulness; SJ = self-judgement; IS = isolation; OI = overidentification; α = Cronbach’s alpha; ω = omega composite reliability; ωh = omega hierarchical; H = construct replicability; ECV = explained common variance; FDI = factor determinacy index. The percent of uncontaminated correlations (PUC) was PUC = .87.

**Table S14:** *Goodness-of-fit indices of the factor models for the Self-Compassion Scale (SCS) and Compassion Scale (CS)*

|  |  | **Exploratory Structural Equation Modelling (ESEM)** | | | |  | **Confirmatory Factor Analysis (CFA)** | | | |
| --- | --- | --- | --- | --- | --- | --- | --- | --- | --- | --- |
| **Scale** | **Model** | **CFI** | **TLI** | **RMSEA (90%CI)** | **WRMR** |  | **CFI** | **TLI** | **RMSEA (90%CI)** | **WRMR** |
|  | One-factor | .875 | .864 | .131 [.126, .136] | 2.161 |  | .839 | .825 | .131 [.127, .136] | 2.270 |
| Self-Compassion | Six-factor | .986 | .975 | .057 [.049, .064] | 0.528 |  | .934 | .925 | .086 [.081, .092] | 1.336 |
| Scale | Hierarchical six-factor | .984 | .974 | .057 [.051, .064] | 0.582 |  | .897 | .886 | .106 [.101, .111] | 1.791 |
|  | Bifactor | .989 | .979 | .051 [.044, .059] | 0.446 |  | .916 | .900 | .099 [.094, .105] | 1.590 |
|  | One-factor | .845 | .822 | .129 [.120, .137] | 1.940 |  | .835 | .810 | .123 [.115, .131] | 1.914 |
| Compassion | Four-factor | .990 | .980 | .043 [.029, .056] | 0.507 |  | .955 | .945 | .066 [.057, .075] | 1.083 |
| Scale | Hierarchical four-factor | .991 | .983 | .040 [.025, .053] | 0.508 |  | .947 | .937 | .071 [.062, .080] | 1.172 |
|  | Bifactor | .995 | .988 | .033 [.013, .049] | 0.396 |  | .961 | .947 | .062 [.055, .075] | 0.994 |

*Note.* ESEM sample = 402, CFA sample = 409. CFI = comparative fit index; TLI = Tucker‒Lewis index; RMSEA = root mean square error approximation; 90% CI = 90% confidence interval of the RMSEA; WRMR = weighted root means square residual. The chosen estimator was diagonally weighted least squares (WLSMV).

**Table S15:** *Compassion Scale (CS) Spanish version*

Por favor, lea cada afirmación cuidadosamente antes de responder. Indique con qué frecuencia se siente o se comporta de la manera indicada en una escala de 1 “Casi nunca” a 5 “Casi siempre”. Responda de acuerdo con lo que realmente refleja su experiencia en lugar de lo que cree que debería ser.

1. Presto mucha atención cuando otras personas me hablan de sus problemas.

2. Si veo a alguien que está atravesando por un momento difícil, trato de cuidarla.

3. No me preocupo por los problemas de otras personas.

4. Sé que todo el mundo se siente deprimido a veces, forma parte de ser humano.

5. Me doy cuenta cuando las personas están molestas, incluso si no dicen nada.

6. Me gusta estar allí para los demás cuando están pasando por dificultades.

7. No pienso mucho en las preocupaciones de los demás.

8. Es importante reconocer que todas las personas tenemos debilidades y que nadie es perfecto.

9. Tiendo a escuchar con paciencia cuando la gente me cuenta sus problemas.

10. Mi corazón está con las personas que son infelices.

11. Trato de evitar a las personas que sufren mucho dolor

12. El sufrimiento es tan solo una parte de la experiencia humana que todos compartimos.

13. Cuando la gente me cuenta sus problemas, trato de mantener una perspectiva equilibrada de la situación.

14. Cuando los demás se sienten tristes, trato de consolarlos

15. No puedo conectar realmente con otras personas cuando están sufriendo

16. A pesar de mis diferencias con los demás, sé que todos sienten dolor igual que yo.

**Table S16:** *Descriptive statistics of the CS and standardised factor loadings for the bifactor exploratory measurement model (n = 811)*

|  |  |  |  |  |  | **λ spec** | | | |
| --- | --- | --- | --- | --- | --- | --- | --- | --- | --- |
| **Items** | ***M (SD)*** | **S** | **K** | ***r_tot_*** | **λ gen** | **I** | **II** | **III** | **IV** |
| **Factor I. Kindness** |  |  |  |  |  |  |  |  |  |
| 2. If I see someone going through a difficult time, I try to be caring toward that person. | 4.36 (0.72) | -.82 | .07 | .58 | .74 | **.35** | -.14 | -.11 | .08 |
| 6. I like to be there for others in times of difficulty. | 4.22 (0.84) | -.87 | .25 | .56 | .65 | **.44** | -.*05* | *.01* | .07 |
| 10. My heart goes out to people who are unhappy. | 3.49 (1.05) | -.24 | -.49 | .39 | .35 | **.43** | .*07* | *-.02* | .10 |
| 14. When others feel sadness, I try to comfort them. | 4.34 (0.73) | -.85 | .23 | .50 | .62 | **.33** | -.*02* | .13 | .*01* |
| **Factor II. Common humanity** |  |  |  |  |  |  |  |  |  |
| 4. I realize everyone feels down sometimes, it is part of being human. | 4.22 (0.96) | -1.11 | .63 | .52 | .33 | *.00* | **.67** | *-.01* | *-.02* |
| 8. I feel it’s important to recognize that all people have weaknesses and no one’s perfect. | 4.65 (0.61) | -1.69 | 2.43 | .44 | .53 | *-.08* | **.49** | *-.02* | *.02* |
| 12. I feel that suffering is just a part of the common human experience. | 4.12 (0.97) | -.94 | .34 | .44 | .29 | *-.03* | **.57** | .14 | *.06* |
| 16. Despite my differences with others, I know that everyone feels pain just like me. | 4.33 (0.91) | -1.35 | 1.45 | .51 | .37 | *.01* | **.64** | .*03* | .*03* |
| **Factor III. Mindfulness** |  |  |  |  |  |  |  |  |  |
| 1. I pay careful attention when other people talk to me about their troubles. | 4.44 (0.73) | -1.15 | .84 | .57 | .93 | -.14 | -.11 | **-.*21*** | -.10 |
| 5. I notice when people are upset, even if they don’t say anything. | 4.16 (0.80) | -.70 | .09 | .35 | .46 | .15 | .10 | **.15** | *.05* |
| 9. I listen patiently when people tell me their problems. | 4.42 (0.74) | -1.21 | 1.25 | .56 | .78 | *.07* | .10 | **-.*06*** | *-.05* |
| 13. When people tell me about their problems, I try to keep a balanced perspective on the situation. | 4.32 (0.76) | -1.04 | 1.18 | .44 | .63 | *-.08* | .07 | **.59** | -.11 |
| **Factor IV. Indifference** |  |  |  |  |  |  |  |  |  |
| 3. I am unconcerned with other people’s problems. | 4.27 (1.08) | -1.48 | 1.36 | .50 | .48 | *-.03* | -.*01* | *-.05* | **.62** |
| 7. I think little about the concerns of others. | 3.84 (1.07) | -.63 | -.40 | .47 | .37 | .08 | .*02* | *.01* | **.58** |
| 11. I try to avoid people who are experiencing a lot of pain. | 3.92 (1.09) | -.80 | -.11 | .36 | .38 | .17 | .*01* | *-.07* | **.34** |
| 15. I can’t really connect with other people when they’re suffering. | 4.20 (1.06) | -1.33 | 1.02 | .50 | .51 | *.04* | .10 | *.03* | **.49** |

*Note.* M = mean; SD = standard deviation; S = skewness; K = kurtosis. r_tot_ = corrected item-total correlations. Standardised factor loadings on the general compassion factor (λ gen) and specific factors (λ spec). Target factor loadings are in bold. Nonsignificant parameters (*p* ≥.05) are italicised.

**Table S17:** *Reliability indices of the bifactor exploratory measurement model for the Compassion Scale (CS)*

| **Scale** | α | ω | ω_h_ | H | ECV | FDI |
| --- | --- | --- | --- | --- | --- | --- |
| General factor | .92 | .92 | .78 | .93 | .58 | .97 |
| K | .70 | .81 | .24 | .42 | .07 | .72 |
| CH | .68 | .80 | .57 | .70 | .17 | .86 |
| MI | .69 | .84 | .02 | .37 | .05 | .80 |
| IND | .68 | .77 | .44 | .61 | .13 | .82 |

*Note.* N = 811; K = kindness; CH = common humanity; MI = mindfulness; SJ = self-judgement; IS = isolation; OI = overidentification; α = Cronbach’s alpha; ω = omega composite reliability; ω_h_ = omega hierarchical; H = construct replicability; ECV = explained common variance; FDI = factor determinacy index. The percent of uncontaminated correlations (PUC) was PUC = .80.

**Figure S2:** *Pearson’s correlations between self-compassion and compassion for others using the different operationalisations (n = 811)*

| **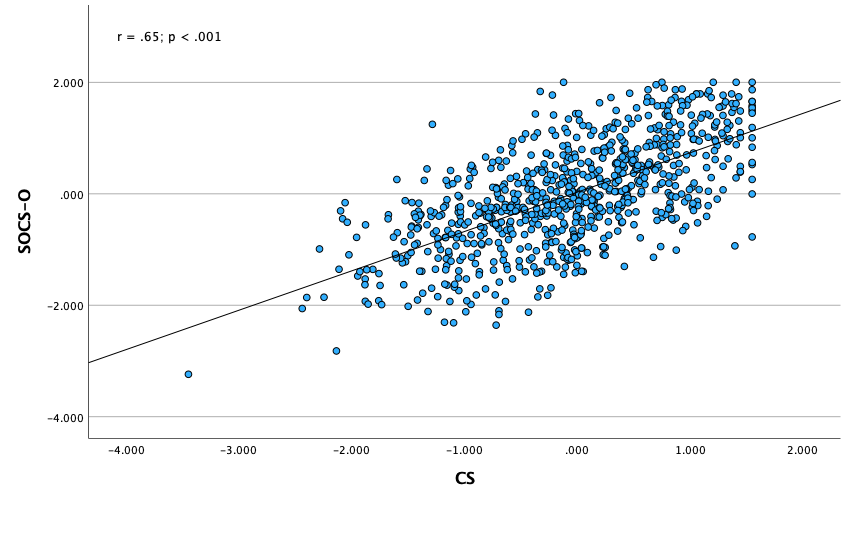** | **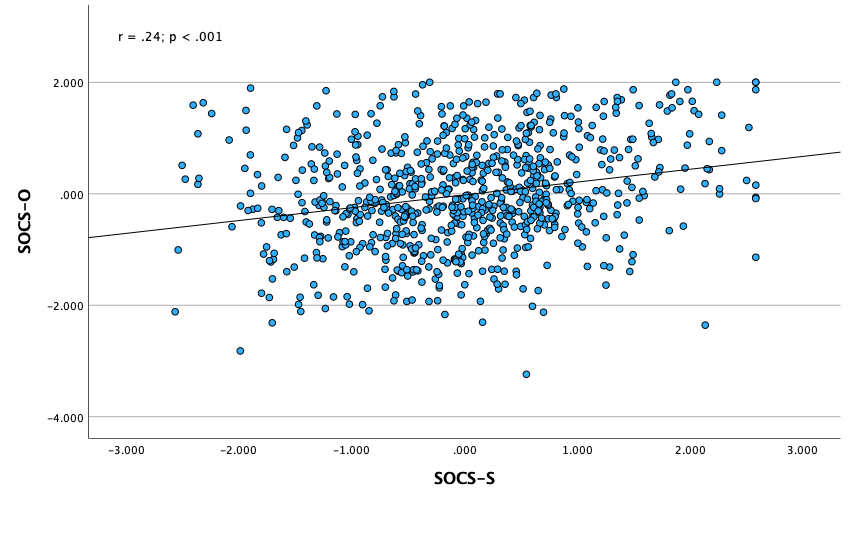** | **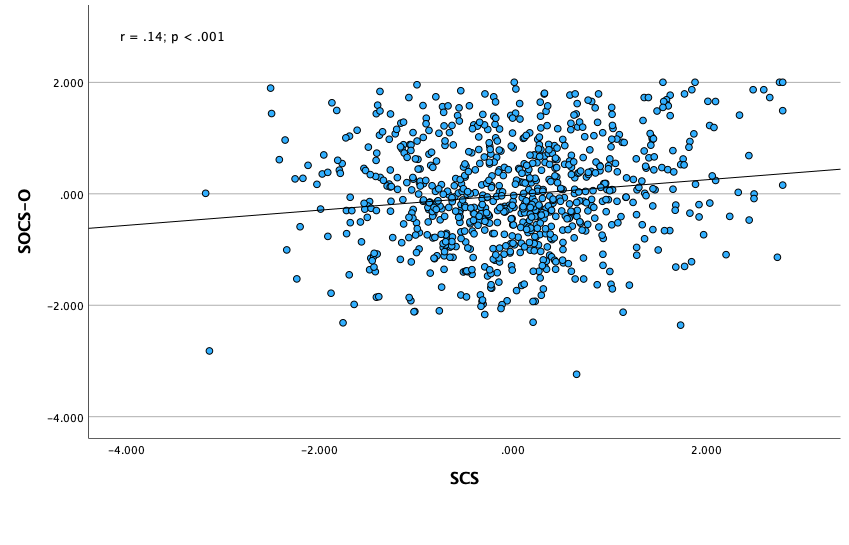** |
| --- | --- | --- |
| **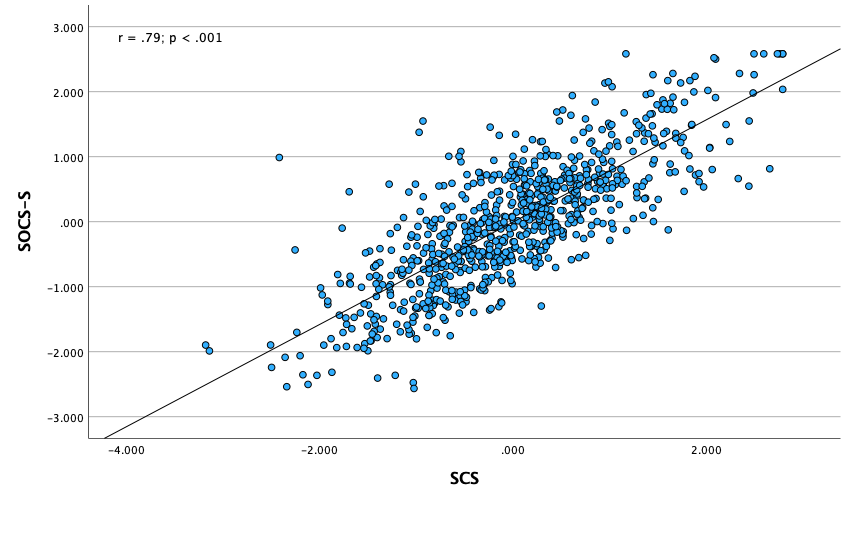** | **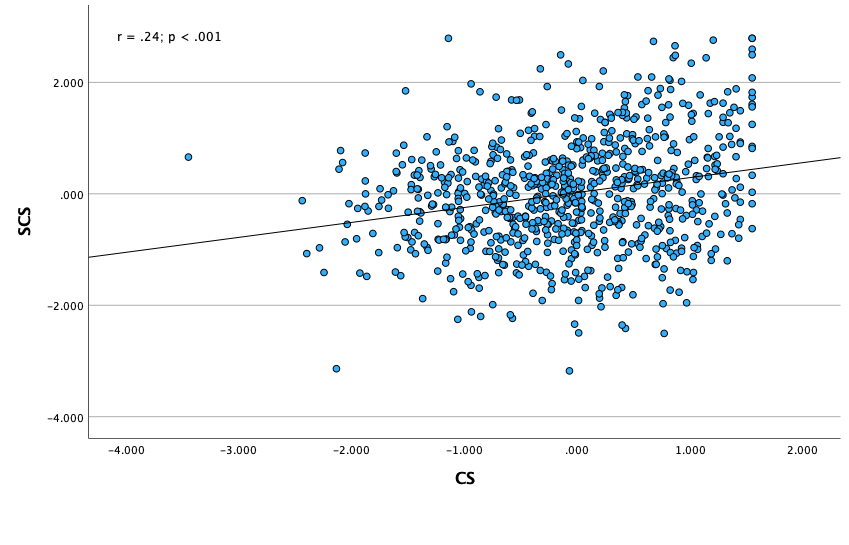** | **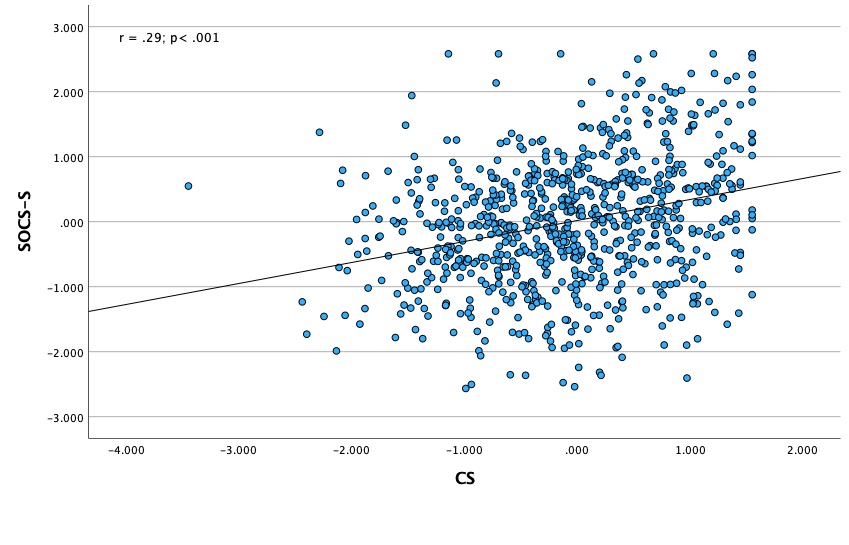** |

*Note.* Graphical representation (scatterplot) of data points involves plotting individual data values on a two-dimensional coordinate system. Each data point is represented by a dot, and its position on the graph is determined by its values on the two variables being plotted. If the points on the graph roughly form a straight line, it indicates a strong linear relationship between the variables. The inclusion of a summary line (i.e., regression line) aims to approximate the overall trend of the data. A positive slope in the regression line suggests a positive correlation, indicating that as one variable increases, the other tends to increase as well. Conversely, a negative slope indicates a negative correlation where one variable increases while the other decreases. Standardised factorial total scores of study variables from bifactor models were used. SOCS-O = Sussex-Oxford Compassion Scale-Others; CS = Compassion Scale (others); SOCS-S = Sussex-Oxford Compassion Scale-Self; SCS = Self-Compassion Scale. r = Pearson’s correlation coefficient.

**Figure S3:** *Pearson’s raw correlations of study variables (n = 811)*

| **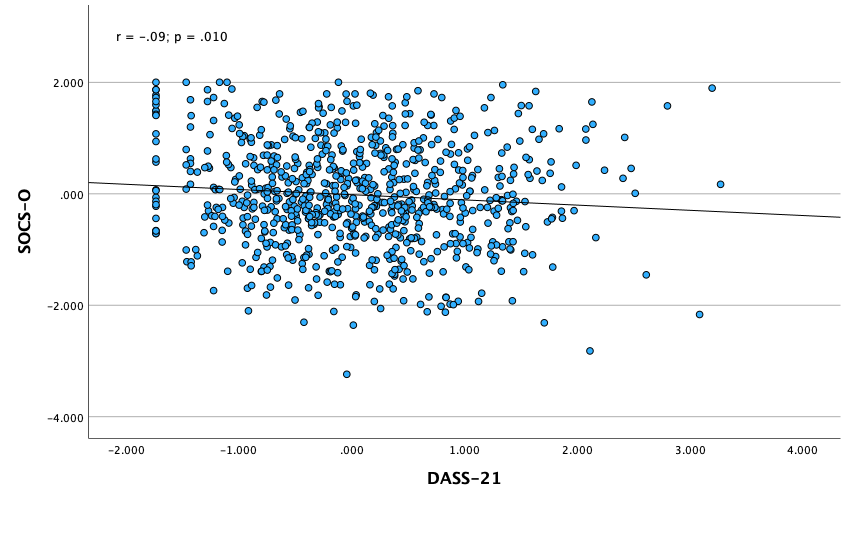** | **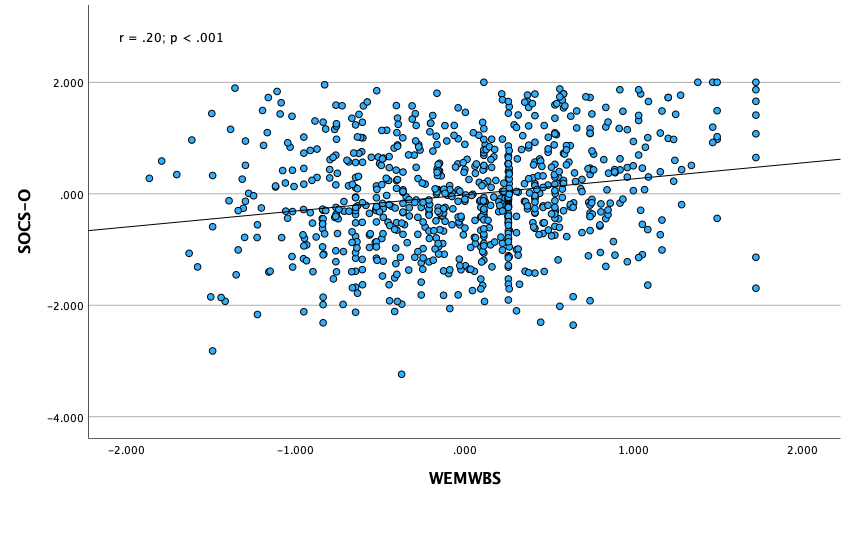** |
| --- | --- |
| **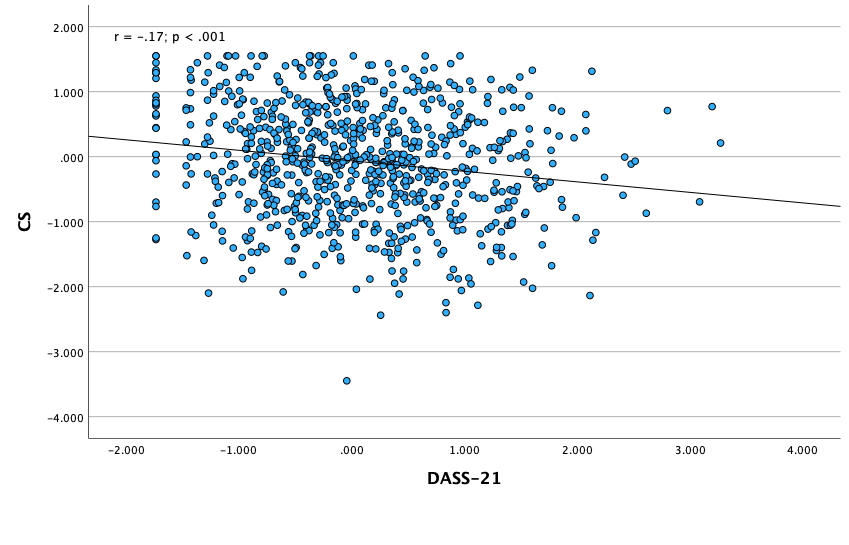** | **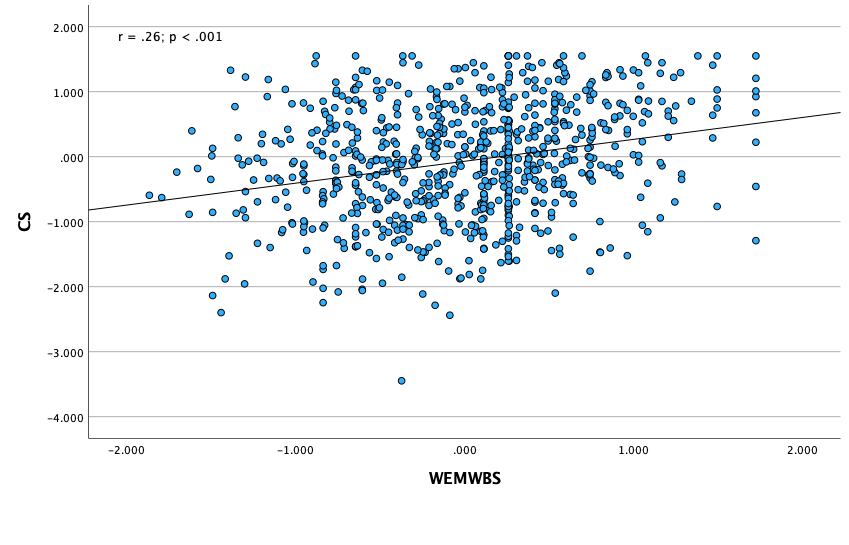** |
| **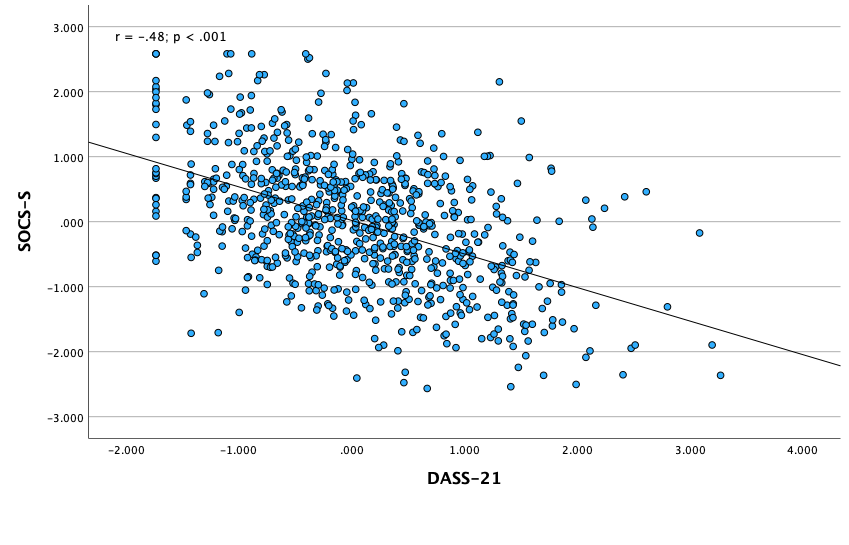** | **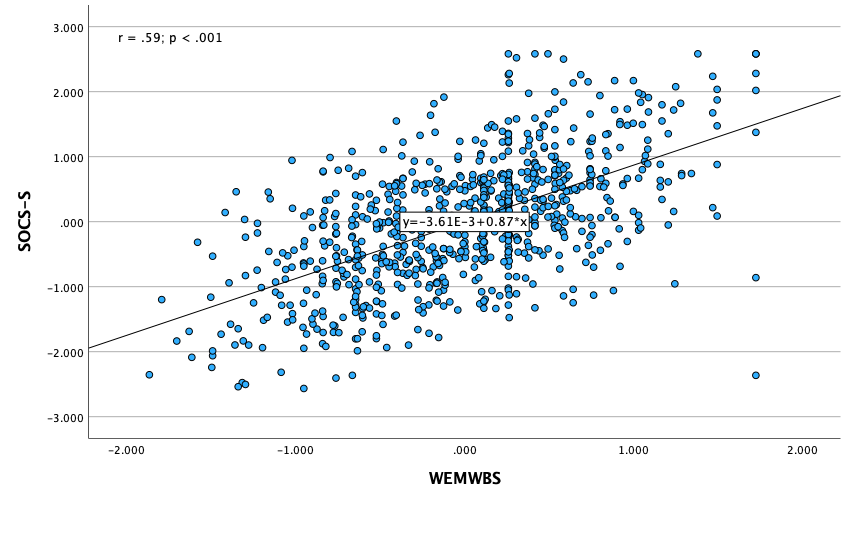** |
| **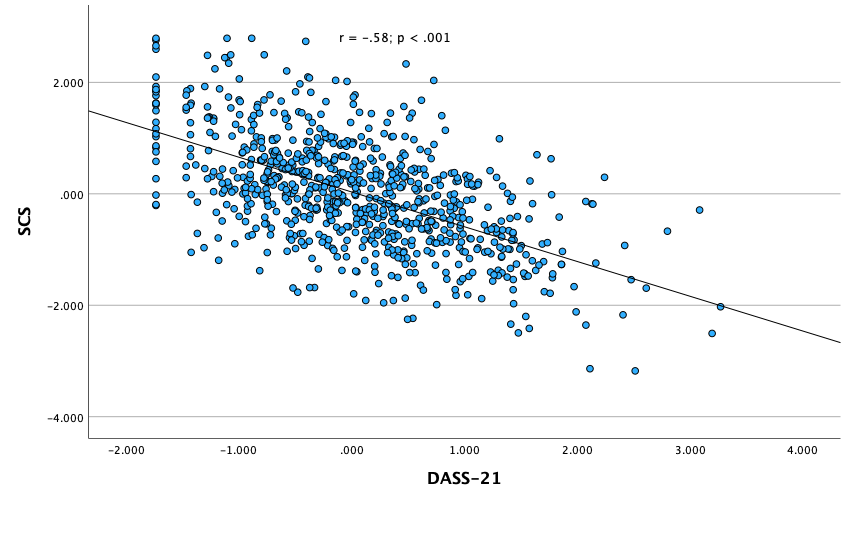** | **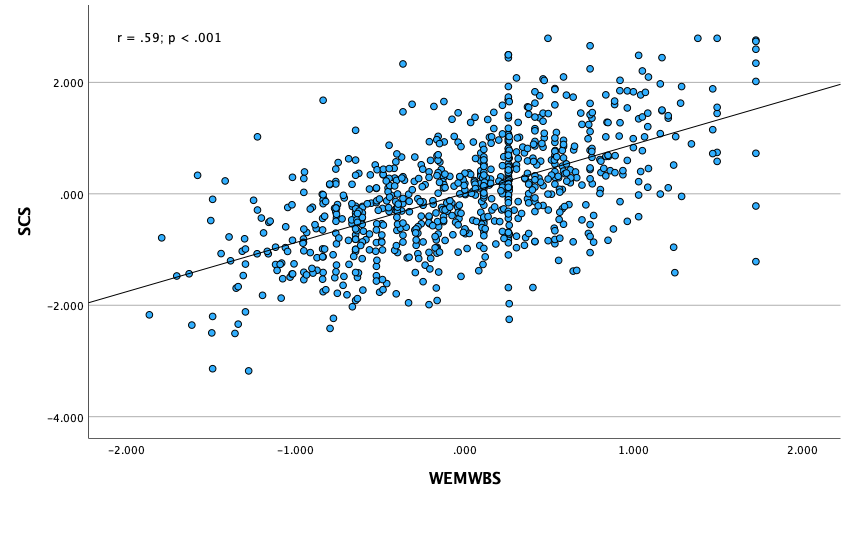** |

*Note.* Graphical representation (scatterplot) of data points involves plotting individual data values on a two-dimensional coordinate system. Each data point is represented by a dot, and its position on the graph is determined by its values on the two variables being plotted. If the points on the graph roughly form a straight line, it indicates a strong linear relationship between the variables. The inclusion of a summary line (i.e., regression line) aims to approximate the overall trend of the data. A positive slope in the regression line suggests a positive correlation, indicating that as one variable increases, the other tends to increase as well. Conversely, a negative slope indicates a negative correlation where one variable increases while the other decreases.. Standardised factorial total scores of study variables were used. SOCS-O = Sussex-Oxford Compassion Scale-Others; CS = Compassion Scale (others); SOCS-S = Sussex-Oxford Compassion Scale-Self; SCS = Self-Compassion Scale. DASS-21 = Depression Anxiety Stress Scale; WEMWBS = Warwick–Edinburgh Mental Well-Being Scale. r = Pearson’s correlation coefficient. DASS-21: M = 14.50 (SD=11.48). WEMWBS: M = 26.32 (SD=3.90).

**Table S18**: *Hierarchical multiple regression analysis predicting DASS-21 with ‘Sociodemographic + SOCSs’ (Step 2)*

| **Step and predictor variable** | | B | 95% CI for B | | | | | | β | *p* | | | | R^2^ | | ΔR^2^ |
| --- | --- | --- | --- | --- | --- | --- | --- | --- | --- | --- | --- | --- | --- | --- | --- | --- |
|  |  |  | LL | | | UL | | |  |  |  |  |  |  |  |  |
|  | |  |  | |  | | | |  |  | | | | .26 | | .19*** |
| Constant | | 0.18 | -0.94 | | 1.30 | | | |  | .752 | | | |  | |  |
| Gender (0 = female; 1 = male) | | -0.24 | -0.38 | | -0.10 | | | | -.11 | .001 | | | |  | |  |
| Age | | -0.00 | -0.01 | | 0.01 | | | | -.01 | .863 | | | |  | |  |
| Number of children | | -0.02 | -0.09 | | 0.05 | | | | -.02 | .550 | | | |  | |  |
| Married | | -0.04 | -0.20 | | 0.12 | | | | -.02 | .639 | | | |  | |  |
| Divorced | | -0.03 | -0.25 | | 0.19 | | | | -.01 | .780 | | | |  | |  |
| Widowed | | -0.07 | -0.48 | | 0.34 | | | | -.01 | .746 | | | |  | |  |
| Primary education | | 0.16 | -0.97 | | 1.29 | | | | .04 | .783 | | | |  | |  |
| Secondary education | | 0.27 | -0.85 | | 1.38 | | | | .10 | .641 | | | |  | |  |
| University degree | | 0.00 | -1.10 | | 1.11 | | | | .00 | .997 | | | |  | |  |
| Unemployed | | -0.16 | -0.45 | | 0.14 | | | | -.04 | .300 | | | |  | |  |
| Employed | | -0.08 | -0.28 | | 0.13 | | | | -.04 | .455 | | | |  | |  |
| Self-employed | | 0.10 | -0.25 | | 0.46 | | | | .02 | .565 | | | |  | |  |
| Homemaker | | -0.06 | -0.63 | | 0.51 | | | | -.01 | .834 | | | |  | |  |
| On a sick leave | | 0.22 | -0.18 | | 0.61 | | | | .04 | .283 | | | |  | |  |
| Retired/pensioner | | -0.11 | -0.48 | | 0.27 | | | | -.03 | .578 | | | |  | |  |
| Unable to work | | -0.16 | -0.65 | | 0.33 | | | | -.02 | .522 | | | |  | |  |
| SOCS-Others | | -0.01 | -0.07 | | 0.06 | | | | -.01 | .853 | | | |  | |  |
| SOCS-Self | | -0.41 | -0.48 | | -0.35 | | | | -.45 | .000 | | | |  | |  |

*Note.* The reference variable for marital status variables is being single. The reference variable for educational level variables is no schooling. The reference variable for employment variables is student. ΔR^2^ is the increment in R^2^ from Step 1 (sociodemoraphic) to Step 2 (sociodemographic + SOCSs).

**Table S19**: *Hierarchical multiple regression analysis predicting SWEMWBS with ‘Sociodemographic + SOCSs’ (Step 2)*

| **Step and predictor variable** | | B | 95% CI for B | | | | | | β | *p* | | | | R^2^ | | ΔR^2^ |
| --- | --- | --- | --- | --- | --- | --- | --- | --- | --- | --- | --- | --- | --- | --- | --- | --- |
|  |  |  | LL | | | UL | | |  |  |  |  |  |  |  |  |
|  | |  |  | |  | | | |  |  | | | | .38 | | .31*** |
| Constant | | -0.69 | -1.74 | | 0.36 | | | |  | .195 | | | |  | |  |
| Gender (0 = female; 1 = male) | | 0.15 | 0.02 | | 0.28 | | | | .07 | .023 | | | |  | |  |
| Age | | 0.00 | -0.01 | | 0.00 | | | | -.03 | .494 | | | |  | |  |
| Number of children | | 0.05 | -0.01 | | 0.12 | | | | .06 | .122 | | | |  | |  |
| Married | | 0.06 | -0.09 | | 0.21 | | | | .03 | .429 | | | |  | |  |
| Divorced | | 0.24 | 0.03 | | 0.44 | | | | .08 | .024 | | | |  | |  |
| Widowed | | -0.03 | -0.41 | | 0.35 | | | | .00 | .875 | | | |  | |  |
| Primary education | | 0.53 | -0.52 | | 1.58 | | | | .13 | .325 | | | |  | |  |
| Secondary education | | 0.47 | -0.57 | | 1.50 | | | | .16 | .380 | | | |  | |  |
| University degree | | 0.57 | -0.46 | | 1.60 | | | | .24 | .278 | | | |  | |  |
| Unemployed | | -0.05 | -0.32 | | 0.22 | | | | -.01 | .725 | | | |  | |  |
| Employed | | 0.13 | -0.06 | | 0.32 | | | | .07 | .173 | | | |  | |  |
| Self-employed | | -0.04 | -0.36 | | 0.29 | | | | -.01 | .830 | | | |  | |  |
| Homemaker | | -0.04 | -0.57 | | 0.49 | | | | .00 | .873 | | | |  | |  |
| On a sick leave | | -0.03 | -0.40 | | 0.33 | | | | -.01 | .858 | | | |  | |  |
| Retired/pensioner | | 0.32 | -0.03 | | 0.67 | | | | .08 | .076 | | | |  | |  |
| Unable to work | | 0.07 | -0.38 | | 0.53 | | | | .01 | .749 | | | |  | |  |
| SOCS-Others | | 0.09 | 0.03 | | 0.14 | | | | .09 | .004 | | | |  | |  |
| SOCS-Self | | 0.52 | 0.46 | | 0.58 | | | | .55 | .000 | | | |  | |  |

*Note.* The reference variable for marital status variables is being single. The reference variable for educational level variables is no schooling. The reference variable for employment variables is student. ΔR^2^ is the increment in R2 from Step 1 (sociodemoraphic) to Step 2 (sociodemographic + SOCSs).

**Table S20**: *Hierarchical multiple regression analysis predicting DASS-21 with ‘Sociodemographic + CS/SCS’ (Step 2)*

| **Step and predictor variable** | | B | 95% CI for B | | | | | | β | *p* | | | | R^2^ | | ΔR^2^ |
| --- | --- | --- | --- | --- | --- | --- | --- | --- | --- | --- | --- | --- | --- | --- | --- | --- |
|  |  |  | LL | | | UL | | |  |  |  |  |  |  |  |  |
|  | |  |  | |  | | | |  |  | | | | .35 | | .28*** |
| Constant | | 0.05 | -1.00 | | 1.09 | | | |  | .928 | | | |  | |  |
| Gender (0 = female; 1 = male) | | -0.18 | -0.31 | | -0.05 | | | | -.08 | .008 | | | |  | |  |
| Age | | 0.00 | -0.01 | | 0.01 | | | | .00 | .965 | | | |  | |  |
| Number of children | | -0.01 | -0.08 | | 0.05 | | | | -.01 | .729 | | | |  | |  |
| Married | | -0.03 | -0.18 | | 0.12 | | | | -.02 | .721 | | | |  | |  |
| Divorced | | -0.01 | -0.22 | | 0.19 | | | | .00 | .889 | | | |  | |  |
| Widowed | | -0.08 | -0.47 | | 0.30 | | | | -.01 | .669 | | | |  | |  |
| Primary education | | 0.28 | -0.77 | | 1.33 | | | | .07 | .603 | | | |  | |  |
| Secondary education | | 0.32 | -0.72 | | 1.35 | | | | .11 | .551 | | | |  | |  |
| University degree | | 0.09 | -0.93 | | 1.12 | | | | .04 | .858 | | | |  | |  |
| Unemployed | | -0.17 | -0.45 | | 0.10 | | | | -.05 | .210 | | | |  | |  |
| Employed | | -0.09 | -0.28 | | 0.11 | | | | -.04 | .383 | | | |  | |  |
| Self-employed | | 0.19 | -0.14 | | 0.52 | | | | .04 | .260 | | | |  | |  |
| Homemaker | | -0.01 | -0.54 | | 0.52 | | | | .00 | .969 | | | |  | |  |
| On a sick leave | | 0.24 | -0.12 | | 0.61 | | | | .04 | .190 | | | |  | |  |
| Retired/pensioner | | -0.15 | -0.50 | | 0.20 | | | | -.04 | .407 | | | |  | |  |
| Unable to work | | -0.16 | -0.61 | | 0.29 | | | | -.02 | .488 | | | |  | |  |
| SOCS-Others | | -0.06 | -0.12 | | 0.00 | | | | -.06 | .051 | | | |  | |  |
| SOCS-Self | | -0.49 | -0.55 | | -0.44 | | | | -.53 | .000 | | | |  | |  |

*Note.* The reference variable for marital status variables is being single. The reference variable for educational level variables is no schooling. The reference variable for employment variables is student. ΔR^2^ is the increment in R2 from Step 1 (sociodemoraphic) to Step 2 (sociodemographic + CS/SCS).

**Table S21**: *Hierarchical multiple regression analysis predicting SWEMWBS with ‘Sociodemographic + CS/SCS’ (Step 2)*

| **Step and predictor variable** | | B | 95% CI for B | | | | | | β | *p* | | | | R^2^ | | ΔR^2^ |
| --- | --- | --- | --- | --- | --- | --- | --- | --- | --- | --- | --- | --- | --- | --- | --- | --- |
|  |  |  | LL | | | UL | | |  |  |  |  |  |  |  |  |
|  | |  |  | |  | | | |  |  | | | | .39 | | .32*** |
| Constant | | -0.43 | -1.47 | | 0.61 | | | |  | .416 | | | |  | |  |
| Gender (0 = female; 1 = male) | | 0.12 | -0.01 | | 0.25 | | | | .05 | .077 | | | |  | |  |
| Age | | 0.00 | -0.01 | | 0.00 | | | | -.03 | .448 | | | |  | |  |
| Number of children | | 0.04 | -0.02 | | 0.10 | | | | .04 | .230 | | | |  | |  |
| Married | | 0.05 | -0.10 | | 0.20 | | | | .03 | .504 | | | |  | |  |
| Divorced | | 0.24 | 0.03 | | 0.44 | | | | .08 | .023 | | | |  | |  |
| Widowed | | -0.01 | -0.39 | | 0.37 | | | | .00 | .961 | | | |  | |  |
| Primary education | | 0.25 | -0.80 | | 1.29 | | | | .06 | .644 | | | |  | |  |
| Secondary education | | 0.24 | -0.79 | | 1.27 | | | | .08 | .651 | | | |  | |  |
| University degree | | 0.33 | -0.69 | | 1.35 | | | | .14 | .521 | | | |  | |  |
| Unemployed | | -0.03 | -0.30 | | 0.24 | | | | -.01 | .853 | | | |  | |  |
| Employed | | 0.15 | -0.04 | | 0.34 | | | | .08 | .120 | | | |  | |  |
| Self-employed | | -0.11 | -0.43 | | 0.22 | | | | -.02 | .512 | | | |  | |  |
| Homemaker | | -0.08 | -0.61 | | 0.44 | | | | -.01 | .761 | | | |  | |  |
| On a sick leave | | -0.12 | -0.48 | | 0.24 | | | | -.02 | .509 | | | |  | |  |
| Retired/pensioner | | 0.39 | 0.04 | | 0.73 | | | | .09 | .030 | | | |  | |  |
| Unable to work | | 0.04 | -0.41 | | 0.49 | | | | .01 | .869 | | | |  | |  |
| SOCS-Others | | 0.15 | 0.09 | | 0.22 | | | | .14 | .000 | | | |  | |  |
| SOCS-Self | | 0.50 | 0.45 | | 0.56 | | | | .53 | .000 | | | |  | |  |

*Note.* The reference variable for marital status variables is being single. The reference variable for educational level variables is no schooling. The reference variable for employment variables is student. ΔR^2^ is the increment in R2 from Step 1 (sociodemoraphic) to Step 2 (sociodemographic + CS/SCS).

| **Table S22:** *Descriptive data and Pearson’s raw correlations between self-compassion and compassion for others per psychological distress and wellbeing subgroups and differences between groups* | | | | | | | |
| --- | --- | --- | --- | --- | --- | --- | --- |
|  |  | CS | | SOCS-S | | SCS | |
|  | *M (SD)* | *r* | *p* | *r* | *p* | *r* | *p* |
| DASS-21≤16 (n = 538) |  |  |  |  |  |  |  |
| SOCS-O | 0.01 (0.92) | .68 | .000 | .30 | .000 | .20 | .000 |
| CS | 0.01 (0.85) |  |  | .33 | .000 | .26 | .000 |
| SOCS-S | 0.26 (0.88) |  |  |  |  | .78 | .000 |
| SCS | 0.31 (0.89) |  |  |  |  |  |  |
| DASS-21>16 (n = 273) |  |  |  |  |  |  |  |
| SOCS-O | -0.07 (0.96) | .61 | .000 | .14 | .018 | .02 | .693 |
| CS | -0.23 (0.86) |  |  | .13 | .039 | .09 | .146 |
| SOCS-S | -0.52 (0.93) |  |  |  |  | .71 | .000 |
| SCS | -0.60 (0.86) |  |  |  |  |  |  |
| DASS-21≤16 vs. DASS-21>16 | *d (95% CI)* | *z* | *p* | *z* | *p* | *z* | *p* |
| SOCS-O | 0.08 (-0.06, 0.23) | 1.61 | .108 | 2.26 | .024 | 2.44 | .014 |
| CS | 0.28 (0.13, 0.43) |  |  | 2.39 | .017 | 2.35 | .019 |
| SOCS-S | 0.87 (0.72, 1.02) |  |  |  |  | 2.12 | .034 |
| SCS | 1.03 (0.88, 1.19) |  |  |  |  |  |  |
|  |  | CS | | SOCS-S | | SCS | |
|  | *M (SD)* | *r* | *p* | *r* | *p* | *r* | *p* |
| SWEMWBS≥28 (n = 354) |  |  |  |  |  |  |  |
| SOCS-O | 0.17 (0.91) | .66 | .000 | .29 | .000 | .23 | .000 |
| CS | 0.18 (0.81) |  |  | .33 | .000 | .29 | .000 |
| SOCS-S | 0.54 (0.86) |  |  |  |  | .76 | .000 |
| SCS | 0.56 (0.92) |  |  |  |  |  |  |
| SWEMWBS<28 (n = 457) |  |  |  |  |  |  |  |
| SOCS-O | -0.17 (0.92) | .62 | .000 | .09 | .068 | -.08 | .076 |
| CS | -0.26 (0.85) |  |  | .09 | .049 | .00 | .999 |
| SOCS-S | -0.42 (0.84) |  |  |  |  | .70 | .000 |
| SCS | -0.42 (0.79) |  |  |  |  |  |  |
| SWEMWBS≥28 vs. SWEMWBS<28 | *d (95% CI)* | *z* | *p* | *z* | *p* | *z* | *p* |
| SOCS-O | 0.37 (0.23, 0.51) | 0.95 | .340 | 2.93 | .003 | 2.17 | .030 |
| CS | 0.53 (0.38, 0.67) |  |  | 3.55 | .000 | 4.20 | .000 |
| SOCS-S | 1.13 (0.99, 1.28) |  |  |  |  | 1.81 | .069 |
| SCS | 1.15 (1.00, 1.30) |  |  |  |  |  |  |

Note. DASS-21 = Depression Anxiety Stress Scale; SWEMWBS = Warwick–Edinburgh Mental Well-Being Scale-Short form; SOCS-O = Sussex-Oxford Compassion Scale-Others; CS = Compassion Scale (others); SOCS-S = Sussex-Oxford Compassion Scale-Self; SCS = Self-Compassion Scale. r = Pearson’s correlation coefficient. z = Steiger’s z for significance of the difference between correlations. M (SD) = mean (standard deviation) using standardised factorial scores. *d* = Cohen’s *d* (effect size measure) when comparing the standardised factorial scores of compassion (SOCS-O, CS, SOCS-S, SCS) according to the subgroups “DASS-21≤16 vs. DASS-21>16” or “SWEMWBS≥28 vs. SWEMWBS<28”. 95% CI = 95% confidence interval.

*References (Supplements)*

Beauducel, A. (2011). Indeterminacy of factor scores in slightly misspecified confirmatory factor models. J. Modern Appl. Statist. Methods 10, 583–598. doi: 10.22237/jmasm/1320120900

Brislin, R.W. (1986). The wording and translation of research instruments. En W. Lonner y J. Berry (Eds.): Field methods in cross-cultural research (pp. 137-164). Beverly Hills, CA: Sage.

Brunner, M., Nagy, G., & Wilhelm, O. (2012). A tutorial on hierarchically structured constructs. Journal of personality, 80(4), 796-846.

DeVellis, R. F. (1991). *Scale development: Theory and applications*. Sage Publications, Inc.

Dillon, W. R., Kumar, A., & Mulani, N. (1987). Offending estimates in covariance structure analysis: Comments on the causes of and solutions to Heywood cases. *Psychological Bulletin,101*(1), 126–135.https://doi.org/10.1037/0033-2909.101.1.126

Garcia-Campayo, J., Navarro-Gil, M., Andrés, E., Montero-Marin, J., López-Artal, L., & Demarzo, M. M. P. (2014). Validation of the Spanish versions of the long (26 items) and short (12 items) forms of the Self-Compassion Scale (SCS). Health and Quality of Life Outcomes, 12(1), 4. https://doi.org/10.1186/1477-7525-12-4

Gorsuch, R. L. (1983). Factor analysis (2nd ed.). Hillsdale, NJ: Erlbaum.

Gu, J., Baer, R., Cavanagh, K., Kuyken, W., & Strauss, C. (2020). Development and psychometric properties of the Sussex-Oxford compassion scales (SOCS). Assessment, 27(1), 3 –20. https://doi.org/10.1177/1073191119860911

Guillemin, F., Bombardier, C., & Beaton, D. (1993). Cross-cultural adaptation of health-related quality of life measures: literature review and proposed guidelines. Journal of clinical epidemiology, 46(12), 1417–1432. https://doi.org/10.1016/0895-4356(93)90142-n

Hancock, G. R., and Mueller, R. O. (2000). “Rethinking construct reliability within latent variable systems,” in Structural Equation Modeling: Present and Future, eds R. Cudek, S. H. C. duToit, and D. F. Sorbom (Lincolnwood, IL: Scientific Software), 195–216.

Hu, L. T., & Bentler, P. M. (1999). Cut-off criteria for fit indexes in covariance structure analysis: Conventional criteria vs. new alternatives. Structural Equation Modeling, 6, 1–55.

Koo, T. K., & Li, M. Y. (2016). A Guideline of Selecting and Reporting Intraclass Correlation Coefficients for Reliability Research. Journal of chiropractic medicine, 15(2), 155–163. https://doi.org/10.1016/j.jcm.2016.02.012

McDonald, R. P. (1999). *Test theory: A unified treatment*. Lawrence Erlbaum Associates Publishers.

Morin, A. J. S., Myers, N. D., & Lee, S. (2020). Modern factor analytic techniques: Bifactor models, exploratory structural equation modeling (ESEM) and bifactor-ESEM. In G. Tenenbaum & R. C. Eklund (Eds.), Handbook of sport psychology (4th ed.). New York, NY: Wiley.

Neff, K. (2003). The development and validation of a scale to measure self-compassion. Self and Identity, 2(793220055), 223–250.

Neff, K. D., Tóth-Király, I., Yarnell, L. M., Arimitsu, K., Castilho, P., Ghorbani, N., … Mantzios, M. (2019). Examining the factor structure of the Self-Compassion Scale in 20 diverse samples: Support for use of a total score and six subscale scores. Psychological Assessment, 31(1), 27–45. https://doi.org/10.1037/pas0000629

Pommier, E., Neff, K. D., & Tóth-Király, I. (2020). The Development and Validation of the Compassion Scale. Assessment, 27(1), 21–39.

Reise, S. P., Bonifay, W. E., & Haviland, M. G. (2013). Scoring and modeling psychological measures in the presence of multidimensionality. Journal of Personality Assessment, 95(2), 129-140.

Rodriguez, A., Reise, S. P., & Haviland, M. G. (2016). Applying bifactor statistical indices in the evaluation of psychological measures. Journal of Personality Assessment, 98(3), 223-237.

Schermelleh-Engel, K., Moosbrugger, H., & Müller, H. (2003). Evaluating the fit of structural equation models: tests of significance and descriptive goodness-of-fit measures. Methods of Psychological Research Online, 8, 23–74.
